# Identification of novel early pancreatic cancer biomarkers KIF5B and SFRP2 from “first contact” interactions in the tumor microenvironment

**DOI:** 10.1186/s13046-022-02425-y

**Published:** 2022-08-24

**Authors:** Harrys Kishore Charles Jacob, Rossana Signorelli, John Lalith Charles Richard, Tyler Kashuv, Shweta Lavania, Ashley Middleton, Beatriz Aguilar Gomez, Anthony Ferrantella, Haleh Amirian, Junyi Tao, Ayse Burcu Ergonul, Melinda Minucci Boone, Marco Hadisurya, Weiguo Andy Tao, Anton Iliuk, Manoj Kumar Kashyap, Monica Garcia-Buitrago, Rajinder Dawra, Ashok Kumar Saluja

**Affiliations:** 1 Department of Surgery, University of Miami, Miller School of Medicine, Miami, USA; 2 Sylvester Comprehensive Cancer Center, University of Miami, Miller School of Medicine, Miami, USA; 3 Department of Biology, University of Miami, Miami, USA; 4 Institute of Engineering in Medicine, University of California San Diego, San Diego, USA; 5 Department of Biochemistry and Molecular Biology, University of Miami, Miami, USA; 6 Department of Pediatrics, Division of Stem Cell Transplantation and Regenerative Medicine, Stanford University School of Medicine, Stanford, USA; 7 Biospecimen Shared Resource, University of Miami, Miami, USA; 8 Department of Biochemistry, Purdue University, West Lafayette, USA; 9 Tymora Analytical Operations, Innovations, West Lafayette, USA; 10 Amity Stem Cell Institute, Amity Medical School, Amity University, Haryana, India; 11 Sylvester Comprehensive Cancer Center, Cancer Modeling Shared Resource, University of Miami, Miller School of Medicine, Miami, USA; 12 Department of Pathology and Laboratory Medicine, University of Miami, Miller School of Medicine, Miami, USA

**Keywords:** Pancreatic Cancer, Extracellular Vesicles, EVtrap, Proteomics, Tumor-Microenvironment, SFRP2, LOXL2, MMP3, KIF5B, Pre-Metastatic Niche, Microbiome, Hybrid TMA, PDAC progression

## Abstract

**Background:**

Pancreatic cancer is one of the most difficult cancers to detect early and most patients die from complications arising due to distant organ metastases. The lack of bona fide early biomarkers is one of the primary reasons for late diagnosis of pancreatic cancer. It is a multifactorial disease and warrants a novel approach to identify early biomarkers.

**Methods:**

In order to characterize the proteome, Extracellular vesicles (EVs) isolated from different *in vitro* conditions mimicking tumor-microenvironment interactions between pancreatic cancer epithelial and stromal cells were analyzed using high throughput mass spectrometry. The biological activity of the secreted EVome was analyzed by investigating changes in distant organ metastases and associated early changes in the microbiome. Candidate biomarkers (KIF5B, SFRP2, LOXL2, and MMP3) were selected and validated on a mouse-human hybrid Tissue Microarray (TMA) that was specifically generated for this study. Additionally, a human TMA was used to analyze the expression of KIF5B and SFRP2 in progressive stages of pancreatic cancer.

**Results:**

The EVome of co-cultured epithelial and stromal cells is different from individual cells with distinct protein compositions. EVs secreted from stromal and cancer cells cultures could not induce significant changes in Pre-Metastatic Niche (PMN) modulation, which was assessed by changes in the distant organ metastases. However, they did induce significant changes in the early microbiome, as indicated by differences in α and β-diversities. KIF5B and SFRP2 show promise for early detection and investigation in progressive pancreatic cancer. These markers are expressed in all stages of pancreatic cancer such as low grade PanINs, advanced cancer, and in liver and soft tissue metastases.

**Conclusions:**

Proteomic characterization of EVs derived from mimicking conditions of epithelial and stromal cells in the tumor-microenvironment resulted in the identification of several proteins, some for the first time in EVs. These secreted EVs cannot induce changes in distant organ metastases in *in vivo* models of EV education, but modulate changes in the early murine microbiome. Among all the proteins that were analyzed (MMP3, KIF5B, SFRP2, and LOXL2), KIF5B and SFRP2 show promise as bona fide early pancreatic cancer biomarkers expressed in progressive stages of pancreatic cancer.

**Supplementary Information:**

The online version contains supplementary material available at 10.1186/s13046-022-02425-y.

## Background

Pancreatic Ductal Adenocarcinoma (PDAC) is a highly aggressive cancer with a survival rate of less than 11% and is expected to become the second leading cause of cancer-related deaths by 2030 [https://www.cancer.org/content/dam/cancer-org/research/cancer-facts-and-statistics/annual-cancer-facts-and-figures/2022/2022-cancer-facts-and-figures.pdf]. The poor prognosis is primarily due to the late presentation of patients with locally advanced tumors at the primary site or an inoperable condition due to distant organ metastases. There is also a lack of biomarkers to identify pancreatic cancers in relatively early pathological stages making it difficult to diagnose the disease before the onset of metastasis. Carbohydrate antigen 19–9 (CA19-9) which is currently the most effective biomarker for pancreatic cancer [1, 2] has a median diagnostic sensitivity of 79% and a median specificity of 80%, which limits its use in the screening of pancreatic cancer [3]. As the validation of biomarkers in a clinical setting is challenging, there is a constant need to develop or revise newer staging systems with biomarkers before being accepted in clinical settings [4–7], making it absolutely essential to identify newer early-stage biomarkers.

Research from Johns Hopkins University indicates that there is a broad window for screening with most diagnoses made after the screening window for pancreatic cancer is closed. It takes nearly a decade for the first cancer-causing mutation to turn into a full-fledged cancer cell and from there on, an average of nearly seven years for the cells to turn into a cancerous mass that has the ability to metastasize to distant sites. Patients die on an average of two and a half years after the onset of distant metastases [8]. This further complicates the sampling timeline where one would investigate the onset of pancreatic cancer. As a novel approach to identify early biomarkers in the development of pancreatic cancer, we chose to examine signaling molecules released from the early Tumor Microenvironment (TME) during primitive conditioning and remodulation. The TME is a complex environment composed of several cellular and protein constituents [9], making it difficult to investigate changes in all of the individual components simultaneously. We chose to investigate early changes induced when activated epithelial cells first come in contact with naïve stromal cells in the TME. This contact initiates and induces signaling pathways in both cell types [10–13], allowing for several proteins to be identified that are released during this “first contact”. We hypothesized that Extracellular Vesicles (EVs) are one of the first mediators released during pancreatic-stromal cross-talk. Previously, our group has shown that PDAC circulating tumor cells (CTCs) secrete EVs that can modulate early neutrophil granulation modifying the immediate microenvironment [14], thus, providing impetus for investigating the “first contact” EVome in the TME.

Investigating TME changes in an animal model of pancreatic cancer is impossible due to difficulty in pinpointing the source of cells that secrete EVs. An isolated system is preferred as we can co-culture individual populations of cells either in isolation or in co-culture conditions. This is a relatively cleaner system to generate and enrich for EVs released due to cancer-stellate cell interactions. A mass spectrometric approach was then used to identify the proteins in the secreted EVome. Biological activity of these secreted EVs in different conditions was assessed for modulating distant organ metastases in two orthotopic models of pancreatic cancer. We also validated key upregulated proteins of significance in both mouse and clinical samples by generating a novel hybrid-TMA. This was eventually followed by analyzing the expression of putative biomarkers in progressive clinical tissue microarrays (TMAs) of pancreatic cancer to ensure that these proteins are robustly expressed both in initiating PanINs (Pancreatic invasive carcinoma and intraepithelial neoplasia) in advanced pancreatic cancer and in nodal and non-nodal metastases.

In this study, we demonstrate a novel strategy to identify early pancreatic cancer biomarkers by using EVs from early “first contact” interactions that drive cancer progression. We used this pipeline to identify KIF5B and SFRP2 as very promising early protein biomarkers secreted by tumor remodulation events associated with cancer cells interacting with naïve stromal cells. These biomarkers are expressed in early PanINs, later stages of pancreatic cancer, as well as in distant organ metastases, making them viable candidates for large-scale screening studies in the future.

## Methods

### Mouse-derived cell lines

KPC cancer cells were isolated from a 5-month-old LSL-Kras^G12D^/LSL-Trp53^R172H^/Pdx1Cre GEMM. Flow cytometric enrichment of CD326^+^/CD31^−^/Fsp1^−^/CD45^−^ cells was performed. The positively enriched cells were cultured in DMEM-F12 medium with 10% FBS. Pancreatic Stellate Cells (PSCs) were isolated according to protocols described earlier [15]. Isolated PSCs were cultured in IMDM with 20% FBS. Cells were cultured in FBS-containing media and shifted to medium containing exosome depleted-FBS prior to the experiment. A total of 10^8 cells were taken in each condition for the KPC cells alone or the PSC cells alone while for the co-culture condition, 9 times the number of PSCs were taken compared to the KPC cells mimicking conditions that are seen *in vivo *[16]*.* Cells were maintained in the exosome-depleted FBS containing media for 24 h and the supernatant was collected and concentrated using a 10KDa filter.

### EV isolation

One hundred ml of cell culture supernatant from all the three cell lines was collected and concentrated using a 10 kDa filter. Ten ml of the concentrate was incubated with magnetic EVtrap beads (Tymora Analytical Operations). The samples were incubated by shaking or end-over-end rotation for 60 min according to the manufacturer’s instructions [17]. The supernatant was removed using a magnetic separator rack, the beads were washed once with PBS, and the EVs were eluted by two 10 min incubations with 100 mM of fresh trimethylamine (TEA, Millipore Sigma). Simultaneously, a fraction of the concentrated supernatant was also subjected to Transmission Electron Microscopy (TEM) and western blot analysis of EV markers.

### Flow cytometric characterization of EVs

The Exo-Flo capture kit was used to characterize the classical markers expressed on the surface of EVs after they were concentrated. CD63, CD81, and CD9 biotin capture antibodies (SBI Bioscience Cat# EXOFLOW150A-1) and anti-Hsp70 biotin-conjugated (Enzo Lifesciences Cat# ADI-SPA-815B-F) were first coupled with magnetic streptavidin beads and were incubated with 1 ml of EVs isolated after concentration. A PBS wash step was done to remove unbound EVs or other contaminants. The cells were stained with ExoFITC (excitation and emission wavelengths of 494 nm and 518 nm) and subjected to flow cytometry. The samples were acquired on a Beckman Coulter CytoFlex S and CytExpert 2.3 was used for acquisition of the data. EVs isolated from PancO2 cell line were used as a published control [18].

### Transmission Electron Microscopy (TEM)

EVs were resuspended in 2% paraformaldehyde and loaded on carbon Formvar-coated copper grids, which were subsequently stained with uranyl acetate. The EVs were fixed overnight in 2% glutaraldehyde in 0.1 M phosphate buffer, post-fixed for 1 h in 2% osmium tetroxide in 0.1 M phosphate buffer, dehydrated through a series of graded ethanols, and embedded in EM-bed (Electron Microscopy Sciences, Fort Washington PA). The glass coverslip was dissolved in hydrofluoric acid. 100 nm sections were cut on a Leica Ultracut EM UC7 ultramicrotome and stained with uranyl acetate and lead citrate. The grids were viewed at 80 kV in a JEOL JEM-1400 transmission electron microscope and images captured using an AMT BioSprint 12 digital camera.

### Preparation of samples for LC–MS

The isolated and dried EV samples were lysed to extract proteins using the phase-transfer surfactant (PTS) aided procedure [17]. The proteins were reduced and alkylated by incubation in 10 mM TCEP and 40 mM CAA for 10 min at 95 °C. The samples were diluted five-fold with 50 mM triethylammonium bicarbonate and digested with Lys-C (Wako) at 1:100 (wt/wt) enzyme-to-protein ratio for 3 h at 37 °C. Trypsin was added to a final 1:50 (wt/wt) enzyme-to-protein ratio for overnight digestion at 37 °C. To remove the PTS surfactants from the samples, the samples were acidified with trifluoroacetic acid (TFA) to a final concentration of 1% TFA, and ethyl acetate solution was added at 1:1 ratio. The mixture was vortexed for 2 min and then centrifuged at 16,000 × g for 2 min to obtain aqueous and organic phases. The organic phase (top layer) was discarded and the aqueous phase was collected, and the step was repeated once more. The samples were dried in a vacuum centrifuge and desalted using Top-Tip C18 tips (Glygen) according to the manufacturer’s instructions. A portion of each sample was used to determine peptide concentration with Pierce Quantitative Colorimetric Peptide Assay, and all samples were normalized based on the total peptide amount. The samples were dried completely in a vacuum centrifuge and the majority of each sample was used for phosphopeptide enrichment using PolyMAC Phosphopeptide Enrichment Kit (Tymora Analytical) according to the manufacturer’s instructions. About 1% of each normalized sample was also injected directly into the LC–MS for proteomics analysis.

### LC–MS/MS analysis

Dried peptide and phosphopeptide samples were dissolved in 4.8 μL of 0.25% formic acid with 3% (vol/vol) acetonitrile and 4 μL of each were injected into an EasynLC 1000 (Thermo Fisher Scientific). Peptides were separated on a 45-cm in-house packed column (360 μm OD × 75 μm ID) containing C18 resin (2.2 μm, 100 Å; Michrom Bioresources). The mobile phase buffer consisted of 0.1% formic acid in ultrapure water (buffer A) with an eluting buffer of 0.1% formic acid in 80% (vol/vol) acetonitrile (buffer B) run with a linear 60- or 90-min gradient of 6–30% buffer B at a flow rate of 250 nL/min. The Easy-nLC 1000 was coupled online with a hybrid high-resolution LTQ-Orbitrap Velos Pro mass spectrometer (Thermo Fisher Scientific). The mass spectrometer was operated in the data-dependent mode, in which a full-scan MS (from m/z 300 to 1,500 with the resolution of 30,000 at m/z 400), followed by MS/MS of the 10 most intense ions [normalized collision energy—30%; automatic gain control (AGC)—3E4, maximum injection time—100 ms; 90 s exclusion].

### MaxQuant label-free quantitation

MS raw files were analyzed using the MaxQuant software [19]. Peptides were searched against the human Uniprot FASTA database using the Andromeda search engine [20], integrated into MaxQuant. Oxidation and N-terminal acetylation, P/T/S phosphorylations were set as variable modifications, while carbamidomethyl was fixed. Trypsin was chosen as the digestion enzyme with a maximum of 2 missed cleavages. Identified peptides had an initial precursor mass deviation of up to 6 ppm and a fragment mass deviation of 0.6 Da. The false discovery rate (FDR) for peptides (minimum of 7 amino acids) and proteins was 1%. A reverse sequence database was used in determining the FDR. For label-free protein quantification, only unique peptides were considered. A contaminant database provided by the Andromeda search engine was used, and all proteins matching the reverse database or labeled as contaminants were filtered out. Label-free protein quantification (LFQ) values were obtained through MaxQuant quantitative label-free analysis [19].

### Data analysis

The abundances of proteins from each group were log (2) transformed and grouped into 3 distinct categories: KPC (cancer cells), PSC (stellate cells), and CoC (co-culture condition). The proteins with detected abundances in at least one category in any condition were taken for analysis. The imputation for the missing abundances was performed by assigning small random values from the normal distribution to each missing value (width = 0.3, down shift = 1.8). Missing values were normally caused by very low abundances. All abundances for each protein were normalized by using width-adjustment. The first, second, and third quartile (q1, q2, q3) were calculated from the distribution of all the values. The second quartile (which is the median) was subtracted from each value to center the distribution and divided by the width in an asymmetric way. All values that were positive after subtraction of the median were divided by q3—q2 while all negative values were divided by q2—q1 following which an ANOVA test was performed. Only those proteins with *q-value* (FDR) less than 0.05 were used in the heatmap. All enrichment analyses with the gene lists were done using GorillaGO [21], an intuitive graphical web application to visualize pathways from different databases. Only pathways that had a *p-value* of ≤ 0.05 were considered. The Reactome [22] database was used for all pathway analyses. All non-human identifiers were converted to their human equivalents, and IntAct interactors were selected to increase the analysis background. Protein datasets were also downloaded from ExoCarta [23] and Vesiclepedia [24, 25] and overlapped with proteins obtained from mass spectrometry to identify bonafide and novel EV proteins.

### EV education and measurement of tumor burden in orthotopic implant models

The following conditions were considered for EV education followed by tumor challenge: Saline only, KPC EVs, PSC EVs, EVs from Co-culture of KPC and PSC cells, and an additional condition in the splenic orthotopic injections, where EVs from the KPC condition were combined with those from PSC cells in a 1:9 ratio. Five µg of EVs were injected into the retro-orbital venous sinus of naïve 6–8 week C57BL6 mice. The injections were done every other day for a period of 25 days. A dual mode of analyzing liver metastases was followed. In the first model, 5000 KPC cells in growth factor reduced Matrigel (Corning Cat# 354,230) were injected orthotopically in the pancreas of mice post-education. The mice were monitored for a period of 4 weeks and were sacrificed. The liver weights were then assessed and compared to WT and saline-treated mice. In the second model, 500,000 KPC cells in growth factor reduced Matrigel (Corning Cat# 354,230) were injected orthotopically in the spleen of mice that received EV injections. The mice were sacrificed 3 weeks post-surgery and the liver weights were measured and compared to WT and saline-control mice. All mice were fed ad-libitum and challenged with tumors except the WT control and monitored according to IACUC guidelines and sacrificed if an excessive deterioration in health was observed.

### Microbiome analyses

#### Fecal collection

Pellets of the fecal matter were collected from five individual mice from each group at 0 time point just before the injection and at two-week time points after education. Stool samples were collected in RNase/DNase-free tubes (Catalog # C-2170, Denville Scientific, Holliston, MA, USA) and were immediately frozen on dry ice and then stored at − 80 °C until further use. The PowerSoil DNA isolation kit (Qiagen Cat #47,016) was used to extract genomic DNA and was stored at -80 °C until amplification. DNA was isolated using DNeasy 96 PowerSoil Pro QIAcube HT Kit with QIAcube HT liquid-handling machine (Qiagen, Maryland, USA).

#### Quantitative real-time PCR amplification for Illumina sequencing

The 16S sequencing was performed at the University of Minnesota Genomic Center [26]. 25ug of DNA was used as templates for PCR amplification of the V4 region of the 16S rRNA gene. Degenerate primer sets were designed with Illumina index sequences on the 5′ end of the reverse primer, which were specific to each fecal DNA sample and allowed for multiplex sequencing. Primers also contained Illumina PCR primer sequences (reverse primer) and Illumina TruSeq Universal Adapter sequences (forward primers) for library creation. The primer sequences (16S-specific portion in bold) used were Meta_V4_515F (TCGTCGGCAGCGTCAGATGTGTATAAGAGACAGGTGCCAGCMGCCGCGGTAA) and Meta_V4_806R (GTCTCGTGGGCTCGGAGATGTGTATAAGAGACAGGGACTACHVGGGTWTCTAAT). The indexing primers are as follows: This step adds both the index and the flow cell adapters. [i5] and [i7] refer to the index sequence codes used by Illumina. The p5 and p7 flow cell adapters are in bold. Forward indexing primer: ATGATACGGCGACCACCGGATCTACAC[i5]TCGTCGGCAGCGTC; Reverse indexing primer: CAAGCAGAAGACGGCATACGAGAT[i7]GTCTCGTGGGCTCGG. PCR reactions were performed using KAPA HiFidelity Hot Start Polymerase. PCR 1 (using the Meta_V4_515F/Meta_V4_806R primer pair): 95 °C 5 min, 20 cycles (98 °C 20 s, 55 °C 15 s, 72 °C 1 min), followed by holding at 4 °C. After the first round of amplification, PCR 1 products were diluted 1:100 and 5 µl of 1:100 PCR 1 was used in the second PCR reaction. PCR 2 (using different combinations of forward and reverse indexing primers): 95 °C 5 min, 10 cycles (98 °C 20 s, 55 °C 15 s, 72 °C 1 min), followed by holding at 4 °C.

#### DNA sequencing

Genomic DNA sequencing was performed using Illumina MiSeq at the University of Minnesota Genomic Center (UMGC). Pooled, size-selected samples were denatured with NaOH, diluted to 8 pM in Illumina’s HT1 buffer, spiked with 15% PhiX, and heat-denatured at 96 °C for 2 min immediately prior to loading. The MiSeq 600 cycle v3 kit was used to sequence the sample. Nextera adapter sequences for post-run trimming were as follows: Read 1: CTGTCTCTTATACACATCTCCGAGCCCACGAGACNNNNNNNNATCTCGTATGCCGTCTTCTGCTTG Read 2: CTGTCTCTTATACACATCTGACGCTGCCGACGANNNNNNNNGTGTAGATCTCGGTGGTCGCCGTATCATT.

#### Sequence processing and analysis

Demultiplexed sequence reads were clustered into amplicon sequence variants (ASVs) with the DADA2 package (version 1.21.0) (27,214,047) implemented in R (version 4.0.3) and RStudio (version 1.1.463). The steps of the DADA2 pipeline include error-filtering, trimming, learning of error rates, denoising, merging of paired reads, and removal of chimeras. The ASV table generated by DADA2 was imported into the QIIME2 pipeline [27] for diversity analyses and taxonomic assignment. Diversity analysis was performed using the Qiime diversity core-metrics-phylogenetic script with a sampling depth of 50,000. Taxonomic assignment of ASVs was done to the genus level using a naïve Bayesian classifier (29,773,078) implemented in QIIME2 with Greengenes reference database (13_8 99%) (22,134,646). LDA Effect Size (LEfSe) (21,702,898) was generated by uploading the taxonomic assignment table to the galaxy app (https://huttenhower.sph.harvard.edu/galaxy/) to detect differentially abundant taxa across groups. The threshold on the logarithmic LDA score for discriminative features was set to 2. Kruskal–Wallis test was used to detect if α diversity differed across treatments. Permutational multivariate analysis of variance (PERMANOVA) was used to detect if β diversity differed across treatments. The Benjamini–Hochberg method was used for controlling the false discovery rate (*q-value*). Bar plot and heat map were generated using microbiomeanalyst (https://www.microbiomeanalyst.ca/) [28].

### Generation of a mouse-human hybrid TMA

A mouse and human-specific TMA was generated for the rapid validation of biomarkers that would need to be assayed. A majority of the samples were taken from the KPC model of pancreatic cancer which is one of the most widely used models for disease modeling [29]. Cores were taken from KPC mice pancreas at various stages of development. Samples were taken from both diseased pancreas and adjacent non-neoplastic areas, as well as from normal pancreas. Distant organ metastases to lungs, liver, or spleen were also cored onto the TMA. Representative sample cores were also taken from subcutaneous tumor models, their associated metastases, and from subcutaneous models of circulating or dissociated tumor cells. Diseased human pancreas along with normal were also taken for assessment in the clinic. Control cores of heart and colon tissue were taken for orientation purposes and to assess staining in other organs. Cores were also taken from an inducible Kras model that was generated by subcutaneously implanting the cells in normal mice. Cores of 1 mm diameter were taken from pathologically verified and arrayed into a TMA with multiple duplicate cores in different locations. The detailed TMA map of all sections arrayed on the hybrid mouse-human TMA is shown in Supplementary File S5.

### Histochemical validation of selected markers

The following antibodies were used for histochemical validation, anti-Loxl2 (Thermo Cat# PA5-85,210; 1:500 dilution), anti-Mmp3 (Thermo Cat# 17,873–1-AP; 1:500 dilution), anti-Sfrp2 (Thermo Cat# PA5-76,794; 1:250 dilution), anti-Kif5b (Abcam Cat# ab167429; 1:500 dilution) and anti-Muc1 (Millipore Sigma Cat#290 M-18). The TMA cores were heated at 60 °C for two hours and then hydrated conventionally, following which antigen retrieval was done by steaming the slides for 20 min in Antigen unmasking solution Tris Based (VectorLabs Cat#H3301-250), following which slides were cooled to room temperature. Endogenous peroxidase, pseudoperoxidase and alkaline phosphatase in FFPE sections were blocked with Bloxall (VectorsLabs Cat # SP6000-100) for 10 min. Cells were then washed in IHC wash buffer (PBS with 0.1% Tween20) for 5 min following which they were incubated with normal goat serum (2.5%) for 20 min for blocking non-specific sites. The antibodies were then diluted in goat serum at the dilutions mentioned earlier and the sections were incubated at 4 °C overnight in a humidified chamber. The slides were then washed in wash buffer for 5 min and incubated for 30 min with ImmPRESS Universal Polymer Reagent (VectorLabs Cat# MP-7451) for 30 min. The slides were washed twice in wash buffer and incubated with ImmPACT DAB EqV peroxidase substrate solution (VectorLabs Cat#SK4103-400) for 5 min. Slides were then washed in wash buffer twice for 5 min each and then rinsed in tap water. The slides were counterstained with Hematoxylin QS counterstain (VectorLabs Cat# H3404-100) for 60 s and rinsed in tap water. The slides were dehydrated conventionally and then mounted with Vectamount permanent mounting medium (VectorLabs Cat# H5000-60). In case of staining for KIF5B on CHTN PancProg1 TMA, the ImmPRESS Duet Double Staining Polymer kit (VectorsLabs Cat#MP-7724) was used with an AP secondary antibody raised against rabbit. The blocking and antibody incubations were done in 2.5% horse serum according to the manufacturer’s instructions. The slides were checked and scored by an expert Pathologist (G-B.M.).

### Pathway mapping for identification of role of KIF5B and SFRP2 in pancreatic cancer

A pathway analysis was performed with the Ingenuity Pathway Analysis (IPA) for SFRP2 and KIF5B. Common pathways involved in pancreatic cancer were taken into consideration to identify networks that could be regulated by overexpression of both proteins. EGF, TGFβ, ERK/MAPK, NOTCH, WNT/β-Catenin, PI3K/AKT, and tTME associated pathways were taken for mapping. First, the path explorer feature was used to find direct as well as indirect paths between KIF5B and SFRP2 and their associated pathways. Following which, the molecule activity predictor (MAP) feature was used to predict the upstream or downstream effects of the upregulated biomarkers.

### Histochemical validation of markers on progression TMA

TMAs were provided by the Cooperative Human Tissue Network which is funded by the National Cancer Institute (Other investigators may have received specimens from the same subjects). The TMA contains representative sections of pancreatic invasive carcinoma and intraepithelial neoplasia (PanIN), including high and low grade pancreatic PanIN and nodal and distant metastases. This TMA represents a very limited number of cases and was used to detect strong trends in differential protein expression. The detailed TMA map and sample information are provided in Supplementary File S5.

## Results

### Characterization of EVs secreted from individual cells and co-culture conditions

**Fig. 1:**
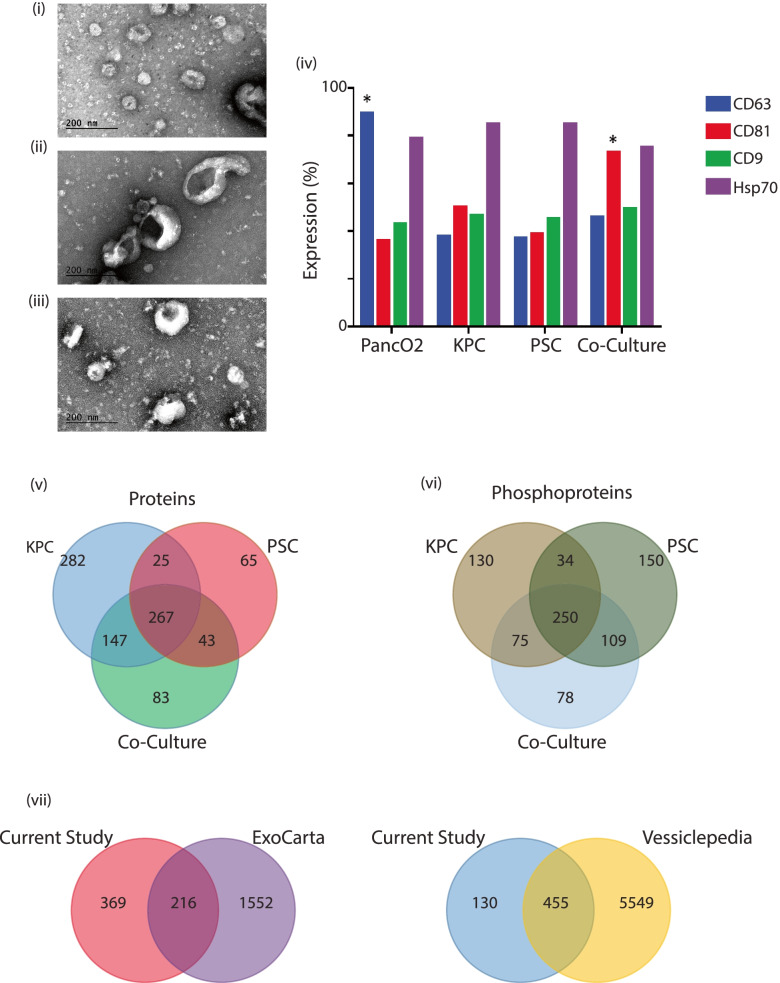
Transmission electron microscopy of EVs isolated from (i) KPC Cells, (ii) PSC Cells and (iii) EVs isolated from KPC and PSC co-culture conditions. All magnifications are shown at 300000X while the scale bar is at 200 nm. (iv) Flow cytometric characterization of CD63, CD81, CD9 EV surface biomarkers and Hsp70 protein in PancO2 cell line and EVs isolated from KPC, PSC and Co-culture conditions. Distribution of overlap of proteins and phosphoprotein identifications from the KPC, PSC and CoC conditions. Distribution is represented as a Venn diagram of (v) proteins and (vi) phosphoproteins. Distribution of proteins represented as a Venn diagram of overlap Exosome and EV databases EcoCarta and Vesiclepedia

Cancer epithelial cells were isolated from a 5-month-old KPC mouse with pancreatic tumors. The cells were sorted to obtain a heterogeneous population of cancer cells that were positive for Epcam and negative for CD45, CD31, and Fsp1 (KPC). We excluded fibroblasts, endothelial cells, and cells of any hematopoietic origin. A heterogeneous population of cells was isolated after sorting and cultured for four passages. Naïve stromal cells (PSCs) were obtained from C57BL6 mice as previously described by Apte et al. [30]. In a pancreatic tumor setting, stromal cells are anywhere from five to ten-fold in excess allowing for us to co-culture cancer and stromal cells in a ratio of 1:9 cells for this study (CoC). Culture supernatant was collected, concentrated, and subjected to transmission electron microscopy (TEM) and flow cytometric analysis to characterize the size and distribution of particles and to help assess purity of the samples. Transmission electron microscopy identified vesicles ranging from 20 nm to greater than 500 nm (Fig. 1 (i-iii)). Additional fractionation was not done to separate vesicles of a particular size, as we wanted to investigate the wholesome contribution of all vesicles to TME remodeling. Conventional vesicles have high concentrations of tetraspanin proteins such as CD63, CD9, and CD81, in addition to Hsp70, which is a pancreatic cancer-specific marker that has also been reported in the exosomal or extravesicular population [31, 32]. A Panc02 line was used as the control pancreatic cell line that is known to secrete EVs [18, 33]. Flow cytometric quantification of EV markers was carried out (Fig. 1 (iv)). The different cell types showed varying concentrations of these assayed proteins (Fig. 1 (iv)) indicating first, an enrichment of EVs and second, the variability in EV composition across the different experimental groups.

### Mass spectrometric characterization of EVome shows a distinct proteome

In addition to conventional proteins characterized on the surface of the EVs, we wanted to investigate the proteome enclosed within these secreted particles. To accomplish this, we collected the supernatant from the different experimental conditions [KPC alone, PSC alone and Co-culture (CoC)]. All samples were processed in triplicate and the purified EVs were subjected to mass spectrometric analysis. Initial proteomic analyses of individual experimental conditions identified 721 proteins from the KPC cells, 400 proteins from the PSCs and 540 proteins from the co-culture condition. Of the identified proteins, 282 were unique to KPC cells, 65 to stellate and 267 in the co-culture condition indicative of the diversity of the different groups providing a milieu for the identification of candidate proteins that regulate or in turn, were regulated in the immediate two-cell TME. Phosphorylation patterns on proteins are indicative of signaling status and activity, hence, we looked at the phosphoproteome of the proteins that are secreted in each cell condition. We identified 489 phosphoproteins in the KPC cell line, 543 in the stellate cells, and 512 in the co-culture condition. Among the proteins, there were 78 phosphorylated proteins that were unique to the co-culture condition, 150 in the stellate PSCs and 130 in the KPC cell line. Venn diagram distributions of proteins and phosphoproteins identified from this study indicate the variability and uniqueness of the EVome detected within the proteome and phosphoproteome datasets (Fig. 1 (v-vi)). The complete list of identifications from the proteomics and phosphoproteomics analyses are shown in Supplementary File S1.

**Fig. 2:**
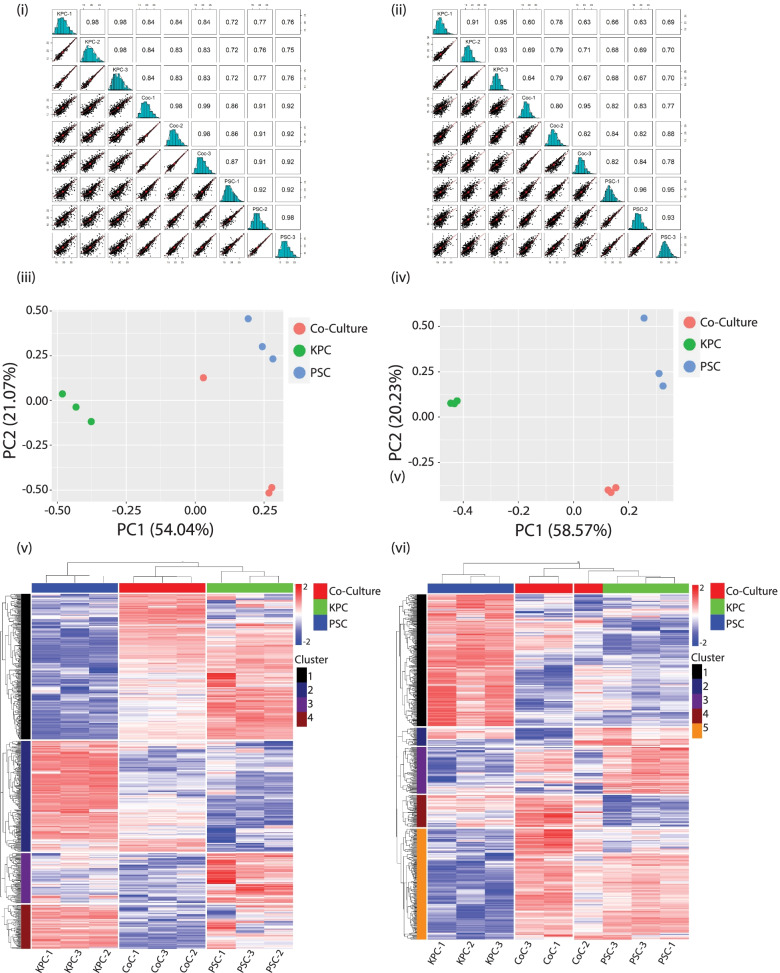
Multiscatter plots of (i) proteins and (ii) phosphoproteins identified from mass spectrometric analyses of the EVome representing correlation between data points obtained from the different experimental groups and biological triplicates among the KPC, PSC and CoC (Co-Culture) conditions. Principal component analyses plot of the (iii) proteins and (iv) phosphoproteins identified to show similarity between samples. Unsupervised hierarchical clustering of (v) proteins and (vi) phosphoproteins identified by mass spectrometry in the triplicate samples of EVs analyzed by mass spectrometry. Distribution of protein expression patterns is classified into clusters with proteins showing similar behavior clustering together

Identifications among the different triplicates of proteins (Fig. 2(i)), presented as multiscatter plots with Pearson correlation values of 0.7 to 1, suggest a good correlation between data obtained from the KPC, PSC, and CoC (Co-culture) experimental conditions. Similarly, correlation values of 0.6–1 were observed among the triplicates of the conditions when phosphoproteins were analyzed (Fig. 2(ii)). PCA plots of the LC–MS data demonstrated that the triplicates were closely clustered among the proteins (Fig. 2(iii)) and phosphoproteins (Fig. 2(iv)) as observed by separation trends between the different conditions. To investigate trends among the candidate proteins and phosphoproteins identified in the study, an unsupervised hierarchical clustering was done. The replicates clustered with each other indicating good reproducibility within each condition. Additionally, we identified four clusters among the proteins indicative of different total protein expression trends (Fig. 2(v)). A more complex trend was observed among the phosphoproteins with five clusters (Fig. 2(vi)). Parallel coordinate plots for protein clusters identified in the study are shown in (Supplementary Fig. 1(i)). Parallel coordinate plots follow the expected trend across clusters, indicative of signature expression patterns within the proteins identified. No observable trends were observed in the parallel coordinate plots for identified phosphoproteins and hence they were excluded for target selection (Supplementary Fig. 1(ii)). Among the different clusters, Cluster 1 included several molecules that were underexpressed in KPC EVs and were overexpressed in CoC and in PSCs. While in cluster 2, the majority of the molecules showed a trend of being overexpressed in the CoC EVs but not in the KPCs and PSCs. Cluster 3 identified the majority of proteins that were overexpressed in PSC and downregulated in KPC and CoC EVomes and finally, cluster 4 showed multiple molecules that were underexpressed in CoC, in comparison to other conditions. The list of proteins that were identified and used in the downstream analyses along with cluster classification is shown in Supplementary File S2.

### Global EV database comparisons identify several common and unique proteins from the EVome

In order to obtain more information about the proteins identified in this study from different experimental conditions, a comparative analysis was done with global databases on extracellular vesicles such as ExoCarta [23] and Vesiclepedia [24, 25]. The common proteins that were used for the analysis among proteins and phosphoproteins is provided in Supplementary File S2. Of the 585 proteins that were identified in this study, nearly 37% (*n* = 216) of them were identified in the ExoCarta database that had 1738 proteins. There were 369 proteins from our dataset that were exclusive to this experiment. We identified nearly 77% (*n* = 455) of the proteins from our study to be reported earlier from Vesiclepedia with a subset of 6004 samples. Among the proteins that were reported in this study, 130 were new identifications that are novel and have not been reported in any of the databases. Figure 1 (vii) shows the distribution of identifications when compared with Vesiclepedia and ExoCarta EV databases.

### Gene Ontology (GO) and reactome analysis of the EVome identifies novel signaling elements

GO analysis of the proteins identified from KPC cells showed that they are mostly enriched for signaling receptor and protein binding molecular functions (actin, growth factor and enzyme); cellular response to organic substances, cytokines, and anatomical structure morphogenesis and development biological processes that are primarily extracellular, cell surface or ECM in their cellular component localizations. Extravesicular proteins from the stellate cells enriched for ECM structural molecules or involved in protein binding molecular functions (SMAD, growth factor, PDGF, and IGF). Common enriched biological processes include skin development, cell and biological adhesion, cellular response to stimulus, and collagen fibril organization. Most proteins were localized to a part of the ECM component or were involved in collagen and fibrillar trimerization. It was interesting to note that 540 proteins identified from the co-culture condition enriched for biological processes that were a cellular response to an organic or chemical stimulus among other response pathways, biological functions involving SMAD, growth factor or PDGF binding for conferring tensile strength to the ECM. A lot of similarity in cellular localization of proteins was observed in KPCs and PSCs. Unique proteins identified in each group were subjected to Reactome analyses to better understand the pathways that could be regulated. Significant pathways enriched in the KPC cells were an association of TriC/CCT chaperonins, which target proteins during biosynthesis and related formation of tubulin folding intermediates, pre-folding mediated transfer of substrate to CCT/Tric, and proteins co-operating with β folding G-protein complexes. Top Reactome pathways enriched for the 65 unique proteins identified in the PSCs were pathways regulating striated muscle contraction, ARMS-mediated activation, defective PGM1, Factor XII, and SERPING1 casing hereditary angioedema, and in collagen degradation among others. The 267 unique proteins identified only in the co-culture conditions enriched for pathways regulating the assembly of collagen fibrils and other multimeric structures and involved in addition to being involved in post-translational protein phosphorylation, collagen biogenesis, degradation, and ECM reorganization. The proteome that was identified in the three conditions were unique and contributed to distinct ECM remodeling capabilities. The detailed lists of all pathways enriched in the GO and Reactome analyses are provided in Supplementary Files S3 and S4.

A Reactome analysis of the unique phosphoproteins identified in the KPC condition enriched for pathways that modulate heparin and heparan sulfate (HS-GAG) degradation and, interestingly, proteins that regulate DNA synthesis on the laggings strand that are involved in gap filling DNA repair or mismatch repair. Phosphoproteins identified in the stellate cells enriched for G2M/M DNA replication checkpoints and for proteins regulating amino acid transport across the plasma membrane and involved in Rhobtb1/2 GTPase cycle regulation. The 250 unique proteins identified in the co-culture condition were enriched for proteins that modulate post-translational phosphorylation, and proteins regulating the Rhobtb GTPase cycle among other pathways. Interestingly, phosphoproteins identified in all three conditions happen to modulate cell signaling and the GTPase cycle in pancreatic cancer, and regulate eukaryotic translation and initiation complexes, indicative of a much more specific role of the phosphoproteome. A list of the pathways enriched in all Reactome analyses is provided in Supplementary File S4.

### EV education does not induce sufficient PMN change to affect liver metastases in two mouse models

**Fig. 3:**
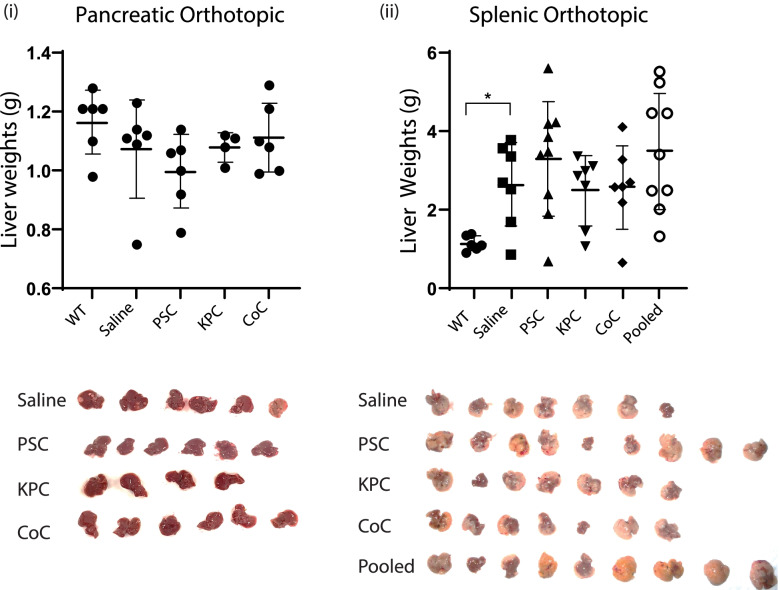
Liver weights (g) of mice that were educated with EVs from PSC, KPC, Co-culture and pooled [KPC: PSC (1:9)] EVs. Controls were treated with saline. WT age matched livers are used as additional controls; (i) Liver weights of mice in pancreatic orthotopic model with representative images of livers with metastases; (ii) Liver weights of mice in splenic orthotopic model with representative images of livers with metastases. This model has additional group with pooled [KPC: PSC (1:9)]EVs

Naïve 6–8 weeks old C57BL6 mice were retro-orbitally injected with 5 μg of EVs to “educate” [34] them and generate a pre-metastatic niche; subsequently, they were challenged with tumors to measure changes in the liver metastases. EVs were concentrated from KPC cancer cells, PSC Stellate cells, and from KPC and PSC cells in co-culture (CoC). An additional condition was also included in the splenic orthotopic model where EVs derived from KPC and PSC cells were mixed in a 1:9 (KPC: PSC) ratio. This would provide information on whether EVs secreted by *in vitro* conditions separately were sufficient to induce an increase in tumor metastasis or if a unique population of EVs were being generated on cell–cell interactions in the TME. After the education period, two models of distant organ metastases were followed. KPC cells in Matrigel were either injected into the pancreas or the spleen. Tumors were allowed to grow for three weeks after which the animals were sacrificed; their livers were necropsied and weighed to measure changes due to KPC cell migration from the different orthotopic sites. Control mice were injected with saline and challenged with tumors. WT mice livers were used as additional age-matched controls. In both models, we observed no statistically significant changes in liver weights among the treatment groups. Experiments were repeated several times with no significant changes observed. Tumor weights from the two different models are provided and representative tumor images provided in Fig. 3 (i) and (ii).

### EVs induce early microbiome changes in EV education models before tumor challenge

**Fig. 4:**
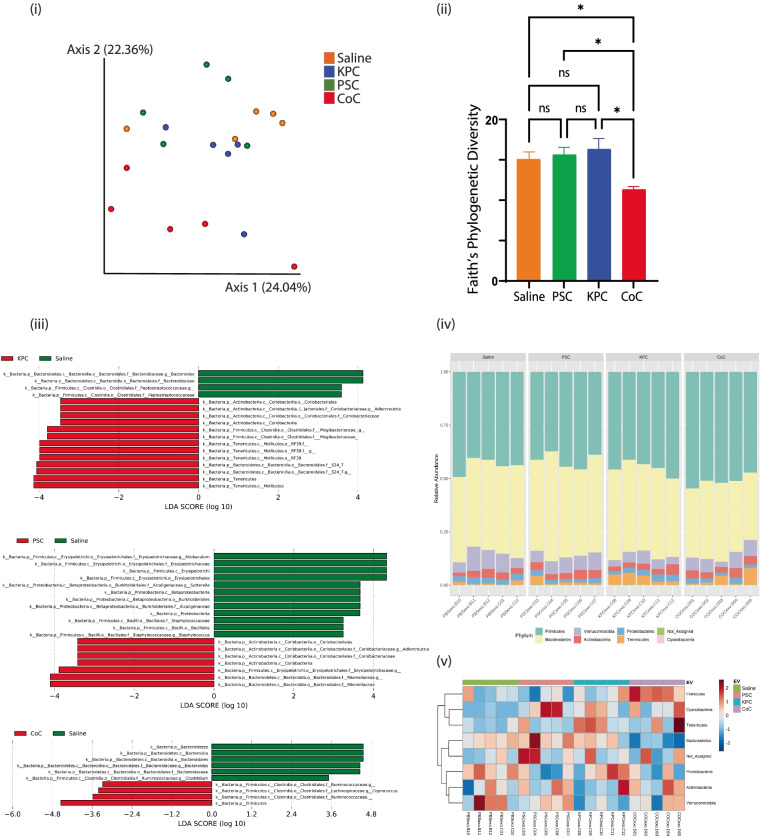
(i) Principal coordinate analysis (PCA) plot of weighted UniFrac distances (metric of β diversity) with q < 0.01 among all four groups (ii) Faith’s phylogenetic diversity (metric of α diversity) at sequencing depth of 19,200; * represents *p* < 0.05; ns represents not significant (iii) LefSe (Linear Discriminant Analysis Effect Size) analysis of bacterial samples among samples KPC Vs PBS control, PSC Vs PBS Control, and CoC (Co-culture) Vs PBS control. The top discriminative bacterial taxa identified between the represented conditions is shown. (iv) Phylum level bacterial composition in the different experimental conditions. Samples include PBS, KPC, CoC and PSC conditions. *N* = 5 samples were considered for the analysis. (v) Heatmap of OTU abundance at the phylum level

To investigate early changes in the gut microbiome of mice that were educated with EVs from different experimental conditions, fecal pellets were collected from mice at the end of two weeks of education. Fecal samples were collected from five mice in each group and DNA was isolated and subjected to microbiome enrichment and analysis. To investigate the differences between microbial communities from the different experimental conditions, β diversity was measured using weighted UniFrac distances and visualized with principal-coordinate analysis (PCoA) plots (Fig. 4 (i)). We observed that the different groups clustered apart, indicating unique bacterial enrichment in each of the experimental groups, and providing additional evidence that the EVs from different groups have a unique influence on the microbiome. Statistical analysis demonstrated that the distinct groups *i.e.*, PBS, KPC, PSC, and CoC are significantly different from each other. The differences are statistically significant, with a measured q value of < 0.05 for all groups. When the intra-sample differences (α diversity) were measured by Faith’s phylogenetic diversity, the CoC microbiome diversity level was significantly lower than that of the other microbiomes (*p* < 0.05) (Fig. 4 (ii)). There were no statistically significant differences between the other groups. It is interesting to note EVs from each of the culture conditions, are selectively poised to induce changes in the mouse microbiome. LefSe (Linear Discriminant Analysis (LDA) analysis was additionally performed between the experimental samples to determine the bacterial taxa that were differentially enriched upon education with different types of EVs. Compared to the PBS control injections, bacteria from family *Coriobacteriaceae*, genus *Adlercreutzia,* were enriched under PSC EV education. In the group educated with EVs from KPC cells, bacteria from the phylum *Tenericutes*, family *Mogibacteriaceae*, genus *Adlercreutzia,* were highly abundant, and bacteria from phylum *Firmicutes,* genus *Coprococcus,* were more abundant in CoC educated samples (Fig. 4 (iii)). Representative bar plots and heat maps of relative abundances at the phylum level are shown in Figs. 4 (iv and v). Consistent with the analysis, phylum *Tenericutes* (orange bar) were more abundant in KPC condition. Similarly, phylum *Firmicutes* being the most abundant than other groups confirmed that it was enriched under CoC condition.

### Immunohistochemical validation of biomarkers identifies novel early biomarkers in pancreatic cancer

We investigated the contribution of EVs either from cancer cells or stellate cells in isolation and finally in a CoC condition. The CoC condition would be identical to *in vivo* conditions where cancer cells interact with stromal cells to generate desmoplastic reaction and cancer progression. The strategy was to investigate if early changes observed in the EV population of cells could help predict and identify novel markers of TME remodeling. Thus, we picked protein candidates that are overexpressed in the CoC conditions but are relatively low or downregulated in the KPC and normal stellate conditions alone. This could be attributed to molecules that are specifically modulated when cancer cells interact with stromal cells. The Cluster with most such molecules was Cluster 2 from the proteins list in Supplementary File S6. A heatmap of protein markers specifically observed in Cluster 2 is shown in Supplementary Fig. 2. The top 20 proteins that were overexpressed in the CoC condition have been shown in Table 1.

**Table 1:** Top 20 proteins identified to be overexpressed in co-culture condition

Gene Symbol	Description	Average PSC Abundance	Average KPC Abundance	Average Co-culture Abundance
*Cxcl2*	C-X-C motif chemokine 2	-0.54722	-0.77428	1.321498
*Mmp3*	MCG9886	-0.83013	-0.48459	1.314718
*Cxcl1*	Growth-regulated alpha protein	-0.5383	-0.77518	1.313477
*Sfrp2*	Secreted frizzled-related protein 2	-0.55275	-0.74882	1.301561
*Kif5b*	Kinesin-1 heavy chain	-0.68642	-0.60435	1.29077
*Crip1*	Cysteine-rich protein 1	-0.47614	-0.79921	1.275346
*Loxl2*	Lysyl oxidase homolog 2	-0.77609	-0.46652	1.242616
*Eef1g*	Elongation factor 1-gamma	-0.41052	-0.81586	1.226383
*Eif5a*	Eukaryotic translation initiation factor 5A-1	-0.59361	-0.62604	1.219655
*Slc9a3r1*	Na( +)/H( +) exchange regulatory cofactor NHE-RF1	-0.6843	-0.51219	1.196483
*Glg1*	Golgi apparatus protein 1	-0.98063	-0.21264	1.193274
*B2m*	Beta-2-microglobulin	-1.01516	-0.16017	1.175321
*Capg*	Capping protein (Actin filament), gelsolin-like	-0.04136	-1.12458	1.165935
*Impdh2*	Inosine-5'-monophosphate dehydrogenase 2	-1.11283	-0.05005	1.162882
*Hdgf*	Hepatoma-derived growth factor	-1.02418	-0.12742	1.151603
*Rbmxl1*	RNA binding motif protein, X-linked-like-1	-0.60948	-0.53024	1.139722
*Il6*	Interleukin-6	-0.05663	-1.08047	1.137099
*Aimp1*	Aminoacyl tRNA synthase complex-interacting multifunctional protein 1	-0.44069	-0.6953	1.13599
*Ccn3*	Protein NOV homolog	0.04454	-1.1511	1.10656
*Lgals4*	Galectin-4	-0.11029	-0.97563	1.085925

We identified several markers that have been previously reported in literature to be involved in TME remodeling, such as Fibronectin, Cxcl2, Cxcl1, and Ccl7. A hybrid TMA was generated using mouse and human normal and diseased pancreas, metastases, KPC GEMM mice organs at different stages, subcutaneous and orthotopic models of pancreatic cancer and tumors and metastases from the EV education experiment described earlier. The hybrid TMA was generated after review from an expert pathologist, and it was arrayed in a format such that there were multiple representations across the slide. Due to the limitations imposed by the availability of antibodies that were reactive to both mouse and human samples, the following biomarkers Kif5b, Sfrp2, Loxl2, and Mmp3 were selected for validation using TMAs. Representative images of tissue sections from mouse and human normal and diseased pancreas were stained by a histological stain Masson’s Trichrome to verify histomorphological differences. Additional staining on subsequent sections for Kif5b, Sfrp2, Loxl2, and Mmp3 was conducted to investigate differences in staining patterns in normal and diseased mouse and human tissue (Supplementary Fig. 3).

#### Lysyl oxidase homolog 2 (Loxl2)

The LOXL family is composed of 5 protein isoforms (LOX and LOXL1-4) that are synthesized as inactive protoenzymes into the extracellular environment and subsequently cleaved into their active form [35], however, our analysis was only focused on the Loxl2 isoform.

**Fig. 5:**
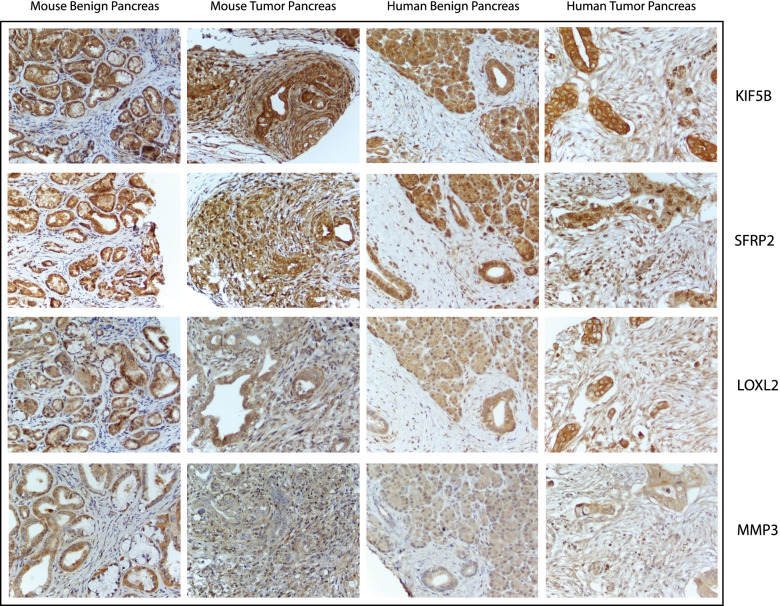
Representative images of mouse and human benign and tumor pancreas at 40X magnification. Stained for KIF5B, SFRP2, LOXL2 and MMP3. Differential expression of the proteins in stroma and tumor are assessed

**Fig. 6:**
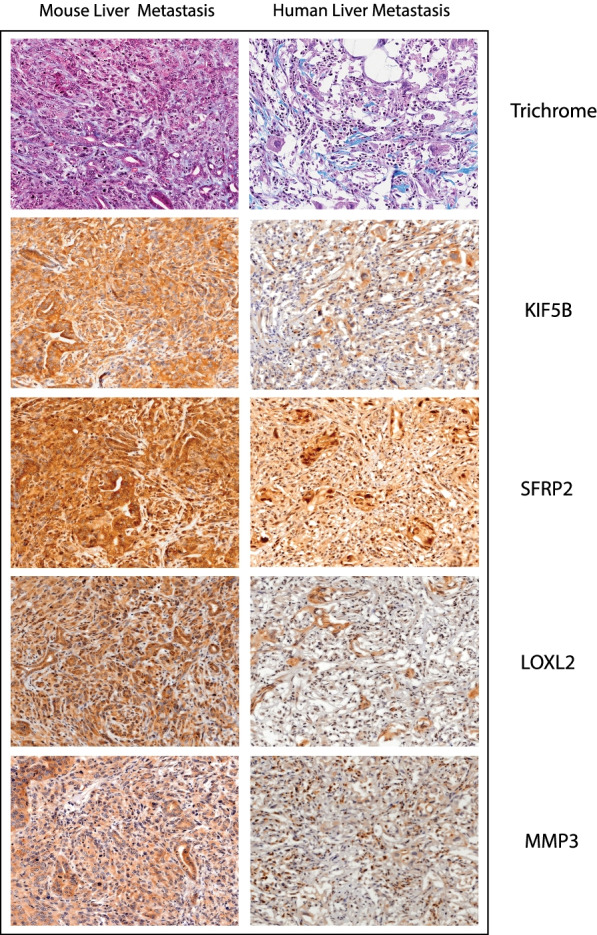
Assessment of biomarker staining in distant liver metastases in mouse and human tumor samples. Histological control Masson’s Trichrome stain and histological staining for markers validated in this study: KIF5B, SFRP2, LOXL2 and MMP3. Image magnifications are at 20X

Loxl2 showed strong expression in the normal pancreatic acini while expression was low in pancreatic ducts and not detected in the stroma. Staining in the diseased pancreas sections showed moderate expression in the murine tumor and stroma, while the human pancreas showed low to moderate expression in the tumor and low expression in the stroma (Supplementary Fig. 3 and Fig. 5**)**. Hepatocytes and pneumocytes stained weakly or undetected for Loxl2 expression. Lung, liver and axillary metastases showed higher expression of Loxl2 in the tumor cells. While no stroma was visible around the lung and axillary metastases, the stroma in the liver mets showed weak staining. Loxl2 expression was also high in metastases observed in mice and human samples. Moreover, it was strongly indicative of the diseased stroma to have been influenced by the cancer cells to secrete EV, which could possibly increase the level of expression in the adjacent stroma (Fig. 6). As for the KPC GEMM mice at different time points, the tumor stained strongly at 25 days, 3 months, and 7 months, indicating that Loxl2 was expressed early in the KPC models, thereby establishing it as an excellent marker for early diagnosis of PDAC in mice (Supplementary Fig. 4). It is also interesting to note that there were distinct differences in the stromal staining where in the same section with diseased and adjacent normal tissue, the stroma around the neoplasia stained strongly for the marker while that around the adjacent normal did not, indicative of highly specific stromal effect in both mouse and human samples (Fig. 6). This indicates that the expression is an outcome of direct exposure of cancer cells to the stroma and is a much-minimized local effect. In terms of consistency in staining across the samples, the staining patterns were not consistent in most cases thereby limiting its utility as a suitable marker for further diagnostic purposes. Circulating and dissociated tumor cells isolated from KPC GEMM mice that were grown subcutaneously were strongly positive for Loxl2 indicating that the cancer cells in circulation also have high expression of Loxl2 protein (Supplementary Fig. 5).

#### Kinesin-1 heavy chain (Kif5b)

The *KIF1B* gene codes for kinesin family member 1B, which is part of the kinesin family of proteins. In neurons, these proteins are responsible for transporting small, sac-like structures called synaptic vesicles whereas in other cell types, these proteins carry the mitochondria. Interestingly, not much is known regarding its expression in the normal or diseased pancreas in either mouse or human samples.

Normal pancreatic acini and ducts stained strongly for Kif5b while the stroma in normal mice and human samples stained weakly (Supplementary Fig. 3 and Fig. 5**)**. Hepatocytes did not or weakly stained while the hepatic ducts showed high expression. The lung alveoli and bronchi also showed overexpression in normal conditions. In the murine pancreas, the protein was overexpressed in both tumor and stroma, while in the human pancreas, the protein expression was low to high in the tumor and low to negative in the stroma (Supplementary Fig. 3 and Fig. 5**)**. Lung, liver, and axillary metastases showed strong positivity in the tumor cells while the stroma in liver mets was weakly stained (Fig. 6). As for the KPC GEMM mice at different ages, the tumor and stroma were strongly positive for expression of Kif5b at 25 days, 3 months, and 7 months. As observed earlier with Loxl2, Kif5b staining in cancer cells and immediate stroma showed a strong positivity while in the normal pancreas, the acini were strongly positive but adjacent stroma was weakly positive for Kif5b expression (Fig. 5). Strong staining was also observed in CTCs and DTCs cultured subcutaneously that were isolated from KPC GEMM mice indicative of it, possibly being a cancer cell secreted factor. Kif5b staining was highly consistent across sample types both for mouse and human and was assayed to be the strongest staining marker among all the markers assayed in our study. Thus, there is a distinct possibility that Kif5b could potentially be a viable and novel marker worthy of being further investigated in pancreatic cancer.

#### Matrix Metalloprotease 3 (Mmp3)

Matrix Metallopeptidase 3, or MMP3, is a protein coding gene; MMP3 protein is identified in exosomes derived from mesenchymal stem cells [36] and is expressed in human bronchoalveolar fluid as well as plasma [37]. Proteins belonging to the MMP family are involved in the breakdown of the ECM in embryonic development, reproduction, tissue remodeling, as well as in metastasis.

Upon evaluation of staining on the hybrid TMA, normal pancreatic acini stained strongly while pancreatic duct weakly stained, and the stroma was negative for Mmp3. Hepatic ducts stained strongly while the hepatocytes were weak or negative for Mmp3. In the lung, alveoli stained weakly while the bronchi were strongly positive for the protein. Mmp3 had low expression in both tumor and stroma in murine and human pancreas (Supplementary Fig. 3 and Fig. 5**)**. Low expression was observed in the lung, liver, and axillary metastases as well (Fig. 6). The KPC GEMMs at different ages also showed weak staining. Interestingly, the only significantly consistent trend was the strong stromal staining in the diseased pancreas; even though acini had strong staining in the normal pancreas, the surrounding stroma was negative for Mmp3 (Supplementary Fig. 4). Mmp3 staining was comparatively weak with respect to all the stains except in the distant metastases (Fig. 6). The pattern of diseased stroma staining more positively than stroma surrounding normal acini was also observed in the hybrid TMAs for both mouse and human tissue (Fig. 5). Weak staining was also observed in both the DTC and CTC subcutaneous implants (Supplementary Fig. 5). To summarize, our results indicate that the Mmp3 was a weakly staining marker, and the staining pattern was not consistent across samples, thereby limiting its diagnostic value as a marker for pancreatic cancer.

#### Secreted frizzled-related protein 2 (Sfrp2)

Soluble frizzled-related proteins, functioning as modulators of Wnt signaling, have major roles to play in regulating the cell growth and differentiation in specific cell types. Specifically, Sfrp2 has a role to play in the retinal development and for myogenesis.

Normal mouse and human pancreatic acini and ducts stained strongly for Sfrp2 while the stroma was negative. Normal murine hepatocytes and hepatic ducts were also strongly positive, while the human hepatocytes and hepatic ducts stained weakly. Normal lung alveoli and bronchi stained strongly as well. The murine tumor and stroma stained strongly in the pancreas while the human tumor staining was variable with weak staining pattern in stromal cells (Supplementary Fig. 3 and Fig. 5). All lung, liver, and axillary metastases were strongly positive in the tumor cells while only stromal cells observed in the liver mets were strongly positive (Fig. 6). In the different ages of KPC GEMM mice, the tumor and stroma strained strongly in the early juvenile mice (Supplementary Fig. 4). The pattern of diseased stroma staining more positively than stroma surrounding the normal acini was also observed in the hybrid TMAs for both mouse and human tissue (Fig. 5). Strong positive staining was also observed in the CTC and DTCs as well (Supplementary Fig. 5). As with Kif5b, Sfrp2 showed a similar consistent staining pattern across different tissues in both the mouse and humans. Thus, our study shows it’s utility as a strongly staining marker which could be used for further diagnostic purposes.

### Overexpression of KIF5B and SFRP2 activates several pancreatic cancer related pathways

**Fig. 7:**
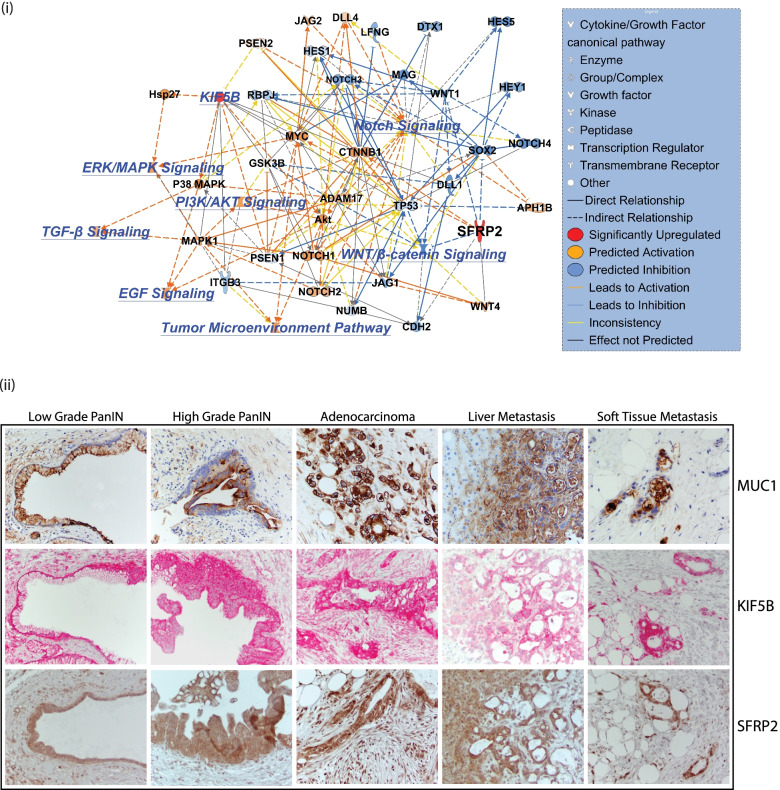
(i) Network mapping of effects of overexpression of KIF5B and SFRP2 affecting signaling pathways in pancreatic cancer. Orange coloring indicates predicted activation while blue coloring indicates predicted inhibition. Solid and broken lines show direct and indirect relationships between the pathway members. Yellow signifies inconsistent relationship. (ii) Expression of MUC1, KIF5B and SFRP2 in low-grade PanIN, high-grade PanIN, adenocarcinoma, Liver metastases, and soft tissue metastases. MUC1 and SFRP2 were stained with DAB-HRP while KIF5B with AP-FastRed. Image magnifications are at 40X

A network mapping of direct and indirect relationships between KIF5B and SFRP2 was done by overlaying their connective map with pathways that are regulated by pancreatic cancer. Predictive overexpression of both KIF5B and SFPRP2 activates pathways that regulate EGF, TGFβ, PI3K/AKT, ERK/MAPK, and Notch signaling while downregulating the WNT/β-Catenin pathway (Fig. 7 (i)). Interestingly, we observed that overexpression of both these proteins downregulates tumor suppressor TP53 which could possibly enhance the tumorogenicity of pancreatic cancer. Additionally, there was a predicted inhibition of RBPC, HES 1 and 5, NOTCH 3 and 4, MAG, WNT1, SOX2, ITGB3, PSEN1, NUMB, CDH2, and JAG1. Predicted upregulation was observed in NOTCH1 and 2, AKT, ADAM17, CTNNB1, MYC, HSP27, P38 MAPK, MAPK1, JAG2, and GSK3B. Based on the predictive modeling, inconsistencies were observed in establishing a link between KIF5B and the WNT/β-Catenin signaling cascade, suggesting additional experiments were needed to verify their relationship. A detailed list of associated molecules and their relationships from Path Designer is provided in Supplementary File S7.

### KIF5B and SFPR2 are expressed in progressive stages of pancreatic cancer

Both Kif5b and Sfrp2 were expressed at higher levels during early stages of co-culture when mouse KPC cells came in contact with naïve mouse stromal cells (PSCs). However, to assess the role of these proteins as early biomarkers in pancreatic cancer it was important to assess their expression in early as well as advancing progressive stages of pancreatic cancer. The CHTN_PancProg1 TMA provided by the Cooperative Human Tissue Network at the University of Virginia was utilized. The TMA has tissue cores with low and high grade intraepithelial neoplasias (PanINs) along with advanced pancreatic cancer, liver and soft tissue metastases. The expression levels of Mucin1 (MUC1) were also used as a control on the progressive TMA. Both SFPR2 and KIFB5 were expressed in early and late stages of pancreatic cancer. Expression was observed in low and high grade PanINs and also in advanced carcinoma. Additionally, expression of these proteins were observed in liver and in soft tissue metastases. Expression was predominantly observed in cancer cells and the surrounding stroma also showed higher expression of the proteins. This was similar to that observed in the mice-human hybrid TMA. The stroma around normal cells was weakly stained for both KIF5B and SFRP2 (Fig. 7(ii)).

## Discussion

Pancreatic cancer has always been associated with diagnosis at advanced stages when treatment options are limited and are always associated with worse clinical outcomes. Most patients tend to be asymptomatic, and when given a late diagnosis, lack therapeutic options. There are currently no biomarkers for the earlier detection of PDAC. We hypothesized that the earliest source of such biomarkers would arise when transformed epithelial cells come in contact with naïve stromal cells present in the stroma, as the EVs released during cancer and stromal cell “first contact” could provide clues in identifying putative biomarkers. EVs have been identified as promising candidates for the identification of biomarkers given their robustness in circulation and in being resistant to degradation [38]. Cancer epithelial cells were isolated from KPC GEMM mice and pancreatic stellate cells (PSCs) from C57BL6 mice. PSCs are resident mesenchymal cells in the pancreas that store lipid droplets and, on activation, secrete factors that stimulate tumor cell growth, cell survival, and metastases [39, 40]. The EVome secreted from individual or CoC conditions was analyzed using mass spectrometry, identifying unique subsets of proteins in all three conditions. We analyzed both the proteome as well the phosphoproteome derived from all three experimental conditions. It is interesting to note that there was a lot of overlap among the three conditions with unique subsets of proteins in all groups. The contribution from the individual cell type could not be identified as we did not label the cells differently and were only analyzing the combined secretome. When compared with global databases such as ExoCarta [36] and Vesiclepedia [24], there was considerable overlap of proteins, and even unique identifications that were exclusive to this study. This is indicative of enrichment of an EV specific proteome by mass spectrometry.

The objective of the study was to investigate only the EVome that was activated when cancer cells came in contact with the stromal cells. Hence, we only investigated proteins that showed relatively lower expression in the KPC and PSC cells, but increased in the CoC conditions. However, prior to validating the expression levels of any of these proteins, we wanted to be certain that the isolated EVome had the biological potency to affect cancer growth or metastases. Research from several groups on the modulation of pre-metastatic niches (PMNs) in pancreatic cancers shows that repeated exposure to EVs from cancer cells affects tumor metastasis in animal models of cancer [18, 34, 41]. Naïve C57BL6 mice were educated with EVs derived from different experimental conditions and were challenged with tumors using two orthotopic models of metastases. We, however, did not find significant changes in liver weights due to increased metastases from the orthotopic site. We postulate that this could be presumably due to the early EVome not being potent enough to induce drastic changes to modulate differences in metastases.

Our focus moved to understanding the proteins that were differentially regulated in the co-culture condition to obtain clues on biological phenomena that could be modulated perhaps by overexpression of certain proteins. We noticed that Secreted Frizzled Related Protein 2 (SFRP2), was among the most upregulated proteins in the CoC condition. The expression of SPRP2 has been shown to be regulated by commensal bacteria such as *Bifidobacterium animalis R101-8* in triple CoC transwell *in vitro* experiments [42]. Additionally, in colorectal cancer, it was observed that the methylation status of SFPR2 was modulated by changes in the microbiome of anthocyanin-treated mice [43]. This finding urged us to question whether the reverse were true; could EVs with higher levels of SFRP2 modulate changes in the gut bacteria? Recent studies on the microbiome indicate that *Bidifidobacterium spp* are progressively enriched in progression of pancreatic cancer in the KC GEMM model [44]. This led us to investigate the microbiome of mice that were educated with EVs before being challenged by tumors. Statistically significant changes were observed among α and β diversities among the different experimental groups. These early EVome changes in the TME could perhaps modulate pancreatic cancer development over time.

To investigate the role of some of the candidate proteins that are overexpressed in the EVs in the co-culture condition, four candidates were selected: KIF5B, SFRP2, LOXL2 and MMP3. A custom TMA was generated with representative cores from mouse and human samples to rapidly assess the importance of the selected biomarkers in mouse models as well as in clinical cases. The hybrid-TMA also contained samples from circulating and disseminated tumor cells isolated from the blood and ascites, respectively. These CTCs were propagated in subcutaneous mouse models after isolating them using the Parsortix CTC isolation system. This TMA was used to rapidly assess expression in mouse and human tissues, circulating cells, and in liver and soft tissue metastases. Expression in all stages would be indicative of the robustness of the marker. All four markers showed similar expression patterns in mouse and human samples. The most distinctive observation was that even though the expression was high in normal acinar cells, the benign stroma did not show expression of the marker. On the other hand, the malignant stroma and cancer cells showed higher expression levels. This could possibly be associated with a very local effect, within the stroma around neoplastic cells mediated by the EVs. LOXL2 and MMP3 were stained weakly and the staining patterns were not consistent across samples, thereby limiting their diagnostic value. This could possibly be associated with the antibody clone that was used; there is a possibility that more specific antibodies could provide clearer and consistent results. Since KIF5B and SFRP2 had consistent staining in benign and tumor tissue as well as in metastases, they can be considered strong markers and incredibly valuable diagnostically.

A network prediction mapping analysis was performed to investigate pancreatic cancer-related pathways that are activated on the activation of both KIF5B and SFRP2. It was interesting to note that several pathways such as the EGF-MAPK, PI3K-AKT, TGFβ, and the TME pathway were some of the activated signaling networks, while the WNT-β Catenin pathway was downregulated. Yet, another interesting finding was that overexpression of these two proteins could downregulate TP53, a tumor suppressor that plays a major role in pancreatic cancer. Additional research is necessary to identify the role of these biomarkers in pancreatic cancer.

Any potency of an early biomarker relies on its expression to be consistent across different stages of cancer progression. A progressive PDAC TMA was also used to assess the status of KIF5B and SFRP2 along with MUC1, which is a routinely used PDAC progression marker [45–47]. These markers were consistently expressed in low grade PanINs, high grade PanINs, and in the primary adenocarcinoma. These proteins were also expressed at higher levels in liver metastases and other soft tissue metastases. This suggests that KIF5B and SFRP2 have great potential to be early biomarkers that are consistently expressed in both cancer mouse models and clinical samples. Additionally, high expression levels were observed in early PanINs, adenocarcinomas, and in distant organ metastases. KIF5B has also been identified both in human plasma and serum samples [48–50] and could possibly be analyzed by liquid biopsy strategies. All these findings suggest that KIF5B and SFRP2 are promising early pancreatic cancer markers. Additional investigations are required in a larger cohort of progressive patient samples to assess its clinical significance and role in pancreatic cancer. The co-culture conditions described in this study, which mimic cellular interactions in the TME can also be extrapolated in other malignancies to discover early cancer biomarkers. Additionally, co-cultures with other cell types could also provide information on different cellular contributions in cancers.

## Conclusions

In summary, our work provides a robust pipeline to identify early biomarkers in pancreatic cancer. In order to identify novel cancer progression biomarkers, our investigation focused on changes in the EVome of isolated cellular components of the tumor microenvironment *in vitro*, followed by an analysis on promising candidates on hybrid and progression TMAs. Proteomic characterization of EVs mimicking “first contact” conditions of tumor-stromal interactions resulted in the identification of KIF5B and SFRP2 as promising early biomarkers that are expressed in progressive stages of pancreatic cancer.

## Supplementary Information

**Additional file 1:Supplementary Figure 1.** Parallel coordinate plots for gene clusters shows 4 clusters among proteins (i) and 5 among phosphoproteins (ii) identified by quantitative mass spectrometric analysis

**Additional file 2:Supplementary Figure 2.** Heat map of average expression values of proteins identified in PSC, KPC and co-culture in Cluster 2 from the heatmap. The proteins are arranged with average values of expression among three replicates in descending order. All 251 proteins are shown and inset shows the top 50 proteins in the pathway. The proteins highlighted in red are candidates that have been validated in this study.

**Additional file 3:Supplementary Figure 3.** Representative cores of mouse and human normal and diseased pancreas from the hybrid TMA. The same sections across different TMAs are shown stained for histomorphological stain. Masson’s Trichrome, followed by staining for protein marker KIF5B, SFRP2, LOXL2 and MMP3. All images are at a magnification of 2.68X.

**Additional file 4:Supplementary Figure 4.** Expression of biomarkers in different developmental stages of KPC GEMM mice. Cores taken at 20X magnification show samples from day 25, 3 months and 7 months after initiation of PDAC in GEMM mice. The sample sections were stained for Kif5b, Sfrp2, Loxl2 and Mmp3

**Additional file 5:Supplementary Figure 5.** Representative TMA sections of dissociated and circulating tumor cells isolated from KPC GEMM mice that were cultured subcutaneously in C57Bl6 mice. The sample sections were stained for Kif5b, Sfrp2, Loxl2 and Mmp3.

**Additional file 6: File S1.** Proteome Discoverer Output of identified proteins and phosphoproteins with comprehensive list of peptides detected by mass spectrometry.

**Additional file 7: File S2.** Clusters identified in the proteome and phosphoproteome in EVs. Only those proteins were considered as identified hits, which had three values in any one triplicate condition.

**Additional file 8: File S3.** GO annotation of proteins and phosphoproteins identified in the mass spectrometric analyses for each experimental condition.

**Additional file 9: File S4.** Reactome analyses of proteins and phosphoproteins identified by mass spectrometric characterization of EVs secreted from cell culture conditions.

**Additional file 10: File S5.** Design and details of donor samples used to construct the Hybrid TMA for rapid validation of biomarkers identified in mass spectrometric studies.

**Additional file 11.**

**Additional file 12.**

## Data Availability

All mass spectrometry and sequencing raw files along with analyses files have been deposited at Biostudies under the accession number S-BSST794.

## Abbreviations

CoC: Co-culture
DMEM: Dulbecco’s Modified Eagles Medium
ECM: Extracellular Matrix
EMT: Epithelial Mesenchymal Transition
EV: Extracellular vesicles
EVome: Extravessicular Proteome
EVtrap: Extracellular vesicles total recovery and purification
FBS: Fetal Bovine Serum
GEMM: Genetically Engineered Mouse Model
KPC: KrasG12D/p53R172H/Pdx1Cre mouse pancreatic cancer GEMM model
MMP: Matrix Metalloproteinase
PDAC: Pancreatic Ductal Adenocarcinoma
PMN: Pre-Metastatic Niche
PSC: Pancreatic Stellate Cell
TME: Tumor Micro Environment
