# Supplementary figures and images for: Identification of novel early pancreatic cancer biomarkers KIF5B and SFRP2 from “first contact” interactions in the tumor microenvironment
Source: J Exp Clin Cancer Res. 2022 Aug 24;41:258. doi: 10.1186/s13046-022-02425-y (PMC9400270; doi:10.1186/s13046-022-02425-y)

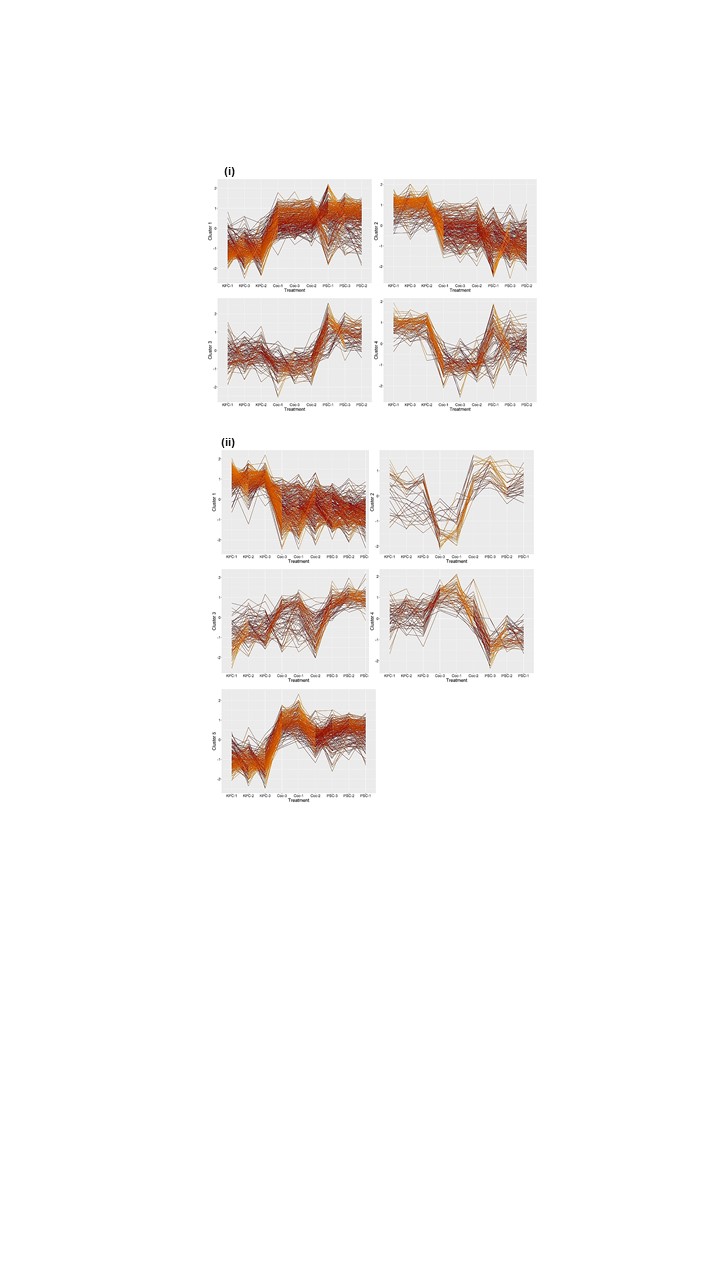

Supplement: Supplementary file 1 — Additional file 1:Supplementary Figure 1. Parallel coordinate plots for gene clusters shows 4 clusters among proteins (i) and 5 among phosphoproteins (ii) identified by quantitative mass spectrometric analysis [file 13046_2022_2425_MOESM1_ESM.jpg]

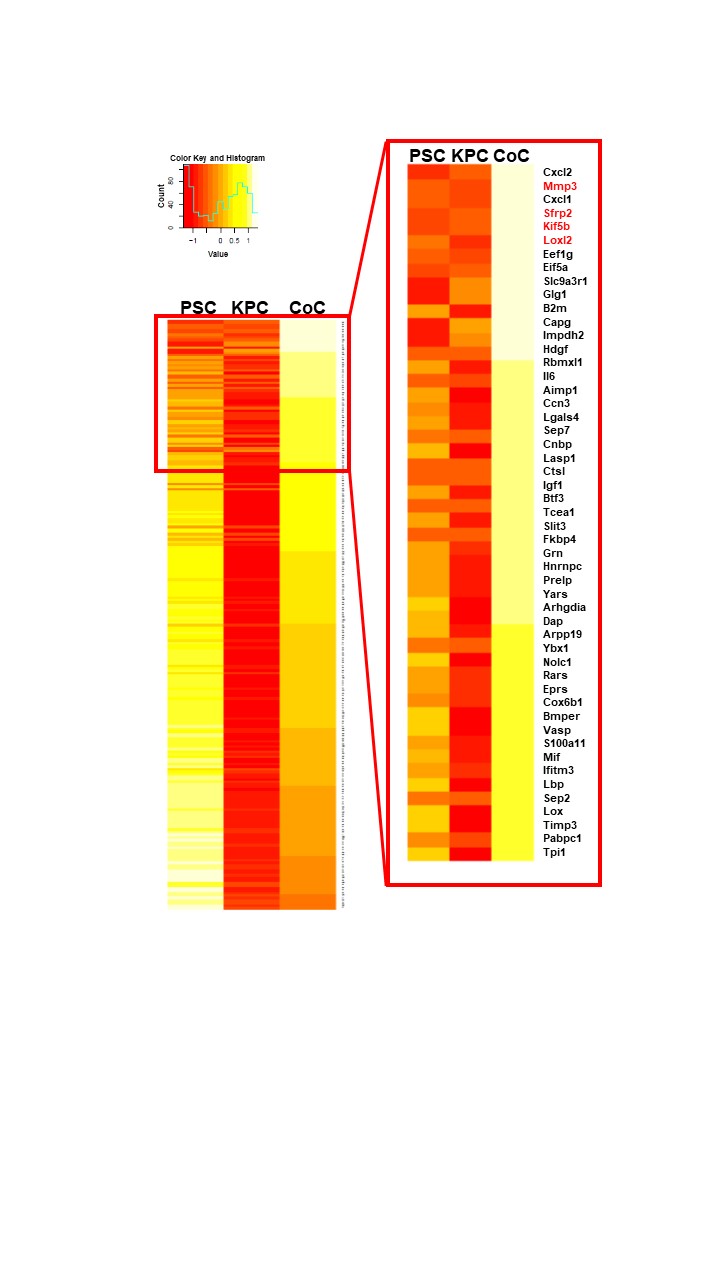

Supplement: Supplementary file 2 — Additional file 2:Supplementary Figure 2. Heat map of average expression values of proteins identified in PSC, KPC and co-culture in Cluster 2 from the heatmap. The proteins are arranged with average values of expression among three replicates in descending order. All 251 proteins are shown and inset shows the top 50 proteins in the pathway. The proteins highlighted in red are candidates that have been validated in this study. [file 13046_2022_2425_MOESM2_ESM.jpg]

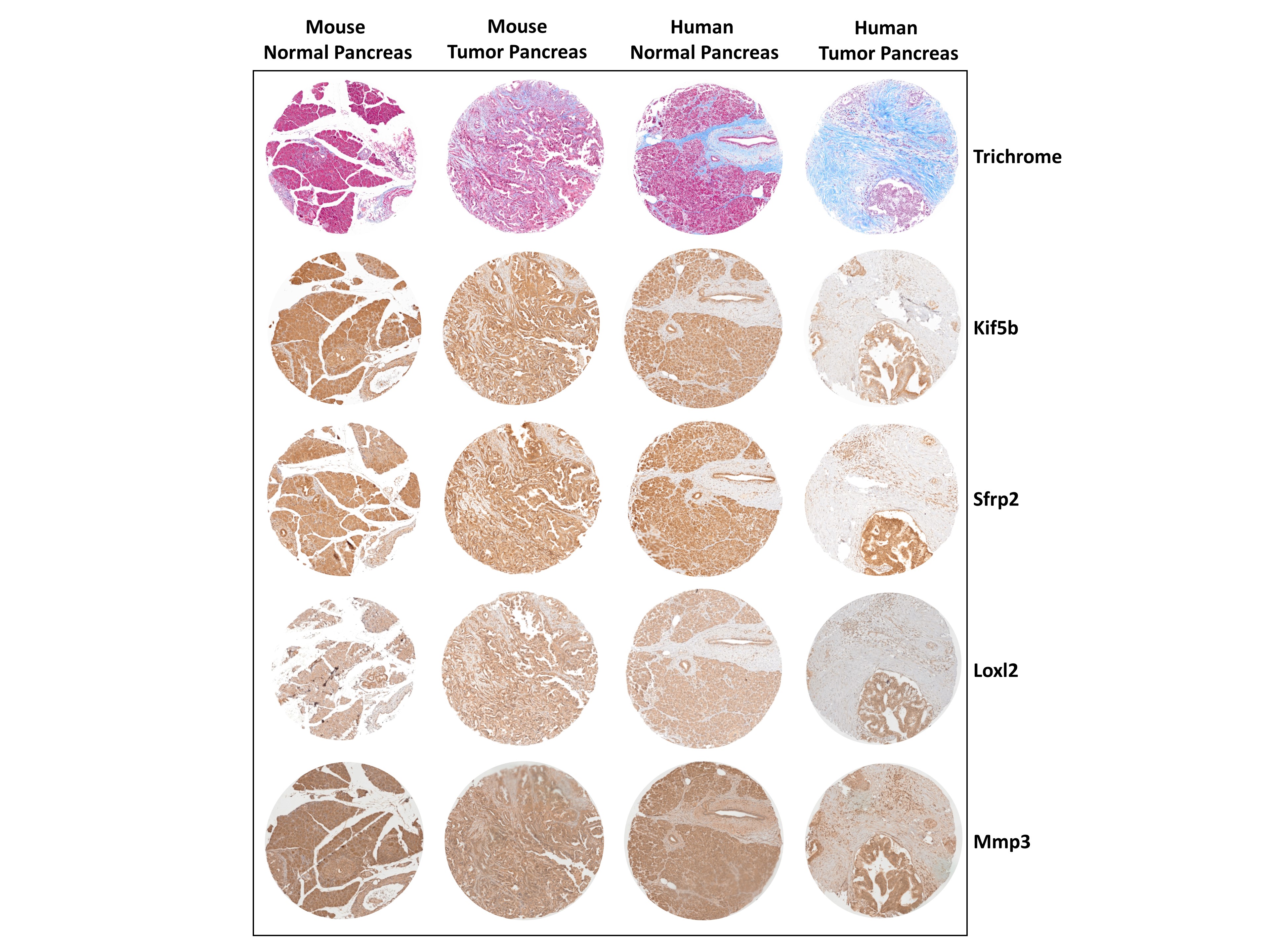

Supplement: Supplementary file 3 — Additional file 3:Supplementary Figure 3. Representative cores of mouse and human normal and diseased pancreas from the hybrid TMA. The same sections across different TMAs are shown stained for histomorphological stain. Masson’s Trichrome, followed by staining for protein marker KIF5B, SFRP2, LOXL2 and MMP3. All images are at a magnification of 2.68X. [file 13046_2022_2425_MOESM3_ESM.jpg]

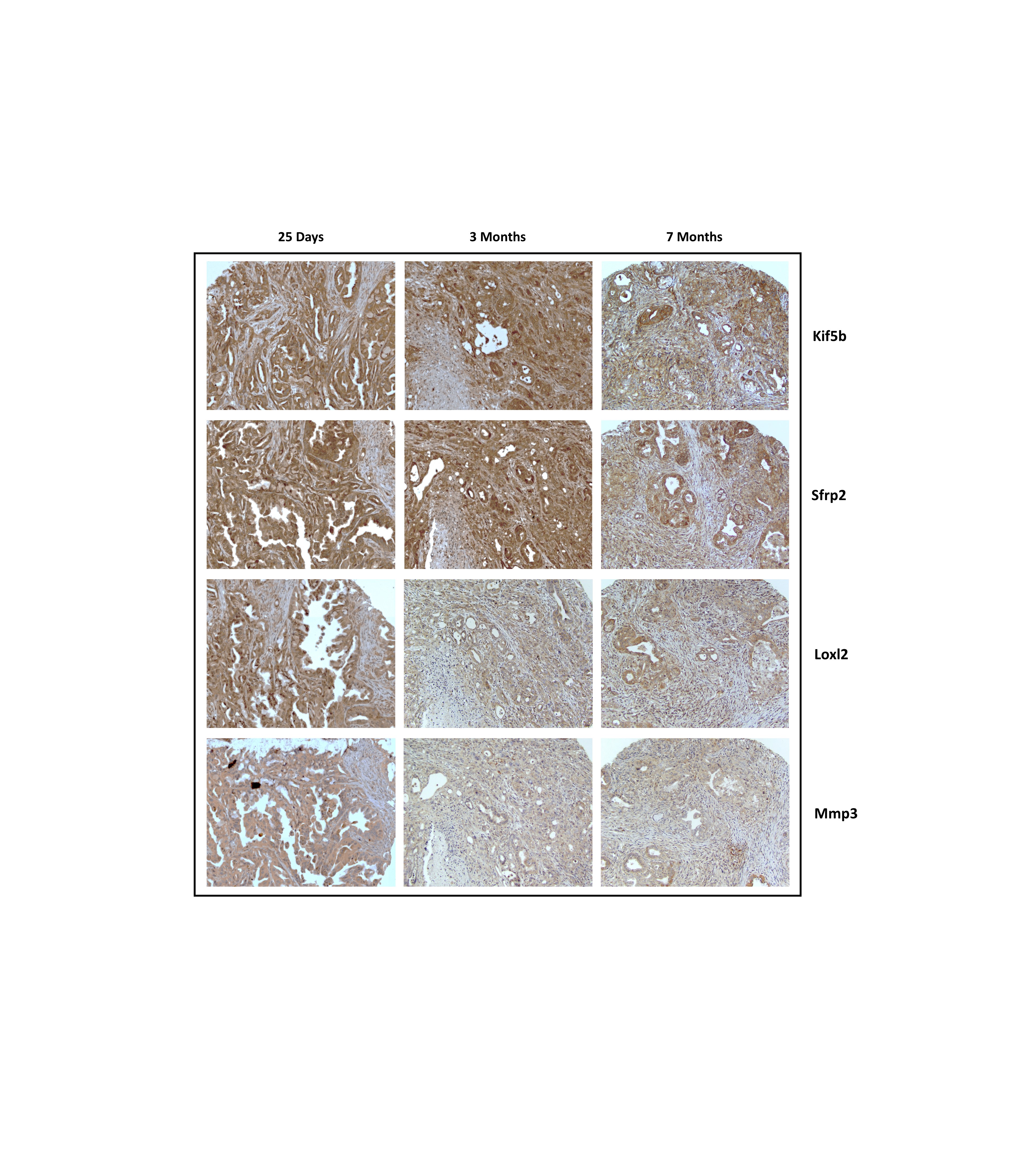

Supplement: Supplementary file 4 — Additional file 4:Supplementary Figure 4. Expression of biomarkers in different developmental stages of KPC GEMM mice. Cores taken at 20X magnification show samples from day 25, 3 months and 7 months after initiation of PDAC in GEMM mice. The sample sections were stained for Kif5b, Sfrp2, Loxl2 and Mmp3 [file 13046_2022_2425_MOESM4_ESM.jpg]

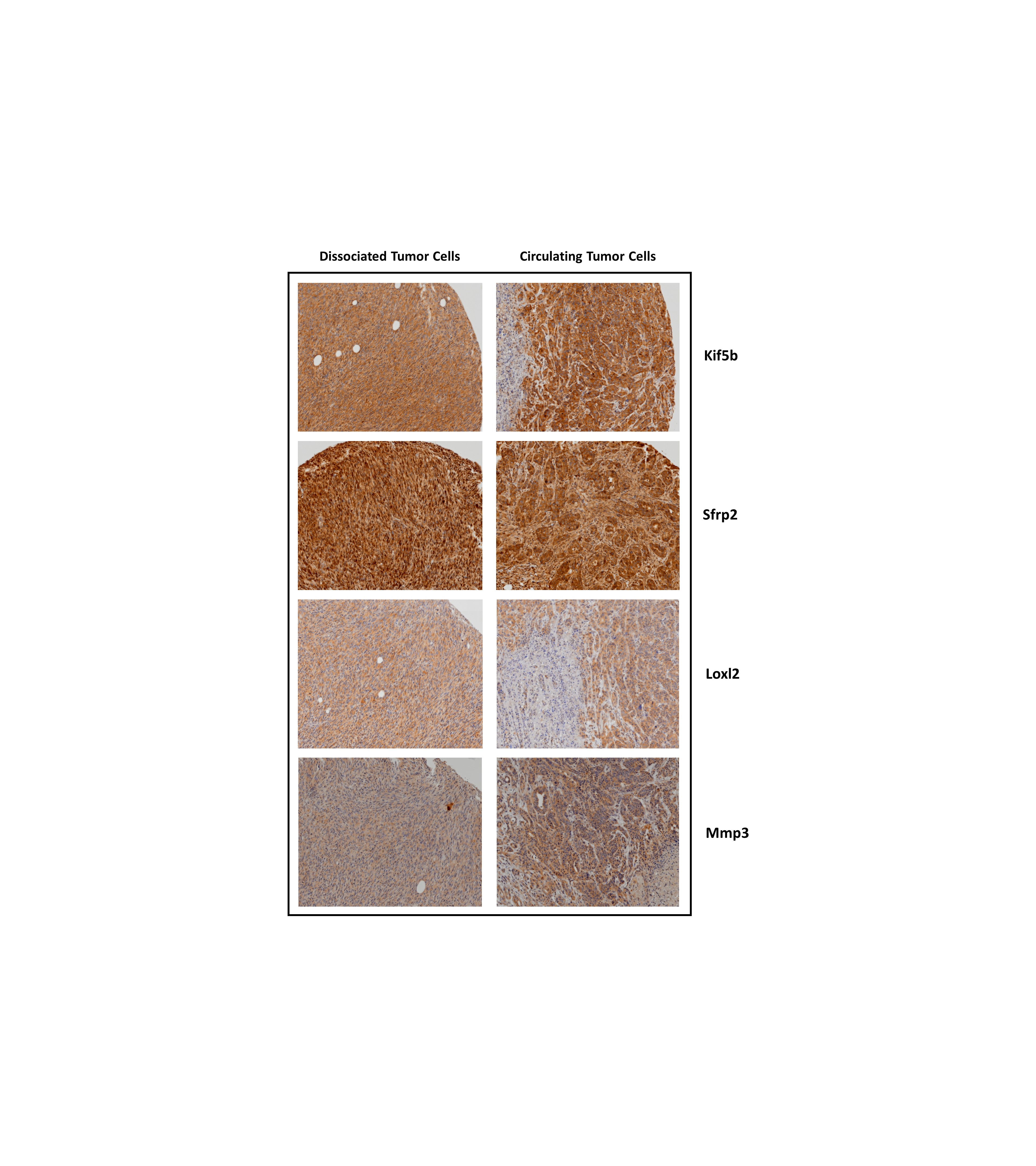

Supplement: Supplementary file 5 — Additional file 5:Supplementary Figure 5. Representative TMA sections of dissociated and circulating tumor cells isolated from KPC GEMM mice that were cultured subcutaneously in C57Bl6 mice. The sample sections were stained for Kif5b, Sfrp2, Loxl2 and Mmp3. [file 13046_2022_2425_MOESM5_ESM.jpg]
