# Supplementary material for: Identification of novel early pancreatic cancer biomarkers KIF5B and SFRP2 from “first contact” interactions in the tumor microenvironment
Source: J Exp Clin Cancer Res. 2022 Aug 24;41:258. doi: 10.1186/s13046-022-02425-y (PMC9400270; doi:10.1186/s13046-022-02425-y)
Supplement: Supplementary file 8 — Additional file 8: File S3. GO annotation of proteins and phosphoproteins identified in the mass spectrometric analyses for each experimental condition. [file 13046_2022_2425_MOESM8_ESM.pdf]

KPC Cancer Cells GO analysis of 721 Identified proteins

Cellular Component

GO term	Description	P-value	FDR q-value	Enrichment (N, B, n, b)
GO:0044421	extracellular region part	4.19E-8	3.49E-5	1.47 (625,215,216,109)
GO:0005576	extracellular region	4.51E-8	1.88E-5	1.52 (625,185,218,98)
GO:0005615	extracellular space	1.87E-5	5.18E-3	1.46 (625,169,216,85)
GO:0009986	cell surface	1.87E-5	3.9E-3	1.99 (625,61,180,35)
GO:0031012	extracellular matrix	3.45E-5	5.74E-3	2.76 (625,101,47,21)
GO:0062023	collagen-containing extracellular matrix	8.14E-5	1.13E-2	1.82 (625,88,160,41)
GO:0043209	myelin sheath	1.37E-4	1.63E-2	2.72 (625,48,91,19)
GO:0005584	collagen type I trimer	4E-4	4.17E-2	48.08 (625,2,13,2)
GO:0048471	perinuclear region of cytoplasm	6.13E-4	5.67E-2	2.53 (625,66,71,19)
GO:0042641	actomyosin	7.52E-4	6.27E-2	8.22 (625,10,38,5)
GO:0016234	inclusion body	8.68E-4	6.57E-2	16.74 (625,4,28,3)

Molecular Function

GO term	Description	P-value	FDR q-value	Enrichment (N, B, n, b)
GO:0005102	signaling receptor binding	3.52E-5	4.21E-2	1.47 (625,127,251,75)
GO:0005515	protein binding	1.41E-4	8.41E-2	1.12 (625,479,248,213)
GO:0051015	actin filament binding	3.06E-4	1.22E-1	2.39 (625,24,185,17)
GO:0003779	actin binding	3.19E-4	9.54E-2	2.08 (625,39,185,24)
GO:0019838	growth factor binding	3.93E-4	9.4E-2	2.30 (625,30,172,19)
GO:0019899	enzyme binding	4.61E-4	9.2E-2	1.38 (625,148,248,81)
GO:0001786	phosphatidylserine binding	5.97E-4	1.02E-1	10.96 (625,6,38,4)
GO:0032557	pyrimidine ribonucleotide binding	8.77E-4	1.31E-1	32.89 (625,2,19,2)
GO:0032551	pyrimidine ribonucleoside binding	8.77E-4	1.17E-1	32.89 (625,2,19,2)
GO:0002135	CTP binding	8.77E-4	1.05E-1	32.89 (625,2,19,2)
GO:0002134	UTP binding	8.77E-4	9.54E-2	32.89 (625,2,19,2)
GO:0001884	pyrimidine nucleoside binding	8.77E-4	8.75E-2	32.89 (625,2,19,2)
GO:0070182	DNA polymerase binding	8.77E-4	8.07E-2	32.89 (625,2,19,2)
GO:0019103	pyrimidine nucleotide binding	8.77E-4	7.5E-2	32.89 (625,2,19,2)
GO:0017098	sulfonylurea receptor binding	8.77E-4	7E-2	32.89 (625,2,19,2)

Biological Processes

GO term	Description	P-value	FDR q-value	Enrichment (N, B, n, b)
GO:0071310	cellular response to organic substance	5.23E-6	3.27E-2	2.10 (625,110,103,38)
GO:0034097	response to cytokine	6.87E-6	2.15E-2	2.84 (625,47,103,22)
GO:0010033	response to organic substance	7.91E-6	1.65E-2	1.90 (625,144,103,45)
GO:0009653	anatomical structure morphogenesis	8.79E-6	1.37E-2	3.09 (625,92,44,20)
GO:0048856	anatomical structure development	1.01E-5	1.26E-2	2.14 (625,176,53,32)
GO:0070887	cellular response to chemical stimulus	1.28E-5	1.33E-2	1.96 (625,127,103,41)
GO:0060351	cartilage development involved in endochondral bone morphogenesis	1.31E-5	1.17E-2	26.04 (625,6,16,4)
GO:0071345	cellular response to cytokine stimulus	2.55E-5	1.99E-2	3.09 (625,40,91,18)
GO:0085029	extracellular matrix assembly	2.72E-5	1.89E-2	15.24 (625,4,41,4)
GO:0032270	positive regulation of cellular protein metabolic process	3.11E-5	1.94E-2	1.40 (625,103,334,77)
GO:0031214	biomineral tissue development	3.48E-5	1.97E-2	24.04 (625,8,13,4)
GO:0045597	positive regulation of cell differentiation	5.61E-5	2.92E-2	2.31 (625,73,100,27)
GO:0032502	developmental process	6.13E-5	2.95E-2	1.76 (625,254,53,38)
GO:0030334	regulation of cell migration	6.33E-5	2.83E-2	1.57 (625,89,251,56)
GO:0051270	regulation of cellular component movement	6.34E-5	2.64E-2	1.54 (625,97,251,60)
GO:2000145	regulation of cell motility	6.36E-5	2.48E-2	1.55 (625,93,251,58)
GO:0017015	regulation of transforming growth factor beta receptor signaling pathway	7.06E-5	2.6E-2	4.75 (625,13,91,9)
GO:1903844	regulation of cellular response to transforming growth factor beta stimulus	7.06E-5	2.45E-2	4.75 (625,13,91,9)
GO:0048518	positive regulation of biological process	7.44E-5	2.45E-2	1.15 (625,310,378,216)
GO:0042221	response to chemical	8.85E-5	2.77E-2	1.73 (625,170,100,47)
GO:0003417	growth plate cartilage development	1.34E-4	3.99E-2	29.30 (625,4,16,3)
GO:0009888	tissue development	1.4E-4	3.98E-2	3.77 (625,49,44,13)
GO:0051240	positive regulation of multicellular organismal process	1.49E-4	4.05E-2	1.81 (625,124,114,41)
GO:0040012	regulation of locomotion	1.57E-4	4.07E-2	1.50 (625,101,251,61)
GO:0051216	cartilage development	1.77E-4	4.42E-2	17.36 (625,9,16,4)
GO:0051247	positive regulation of protein metabolic process	1.84E-4	4.43E-2	1.36 (625,109,334,79)
GO:1900180	regulation of protein localization to nucleus	1.87E-4	4.32E-2	4.96 (625,12,84,8)
GO:0016049	cell growth	1.92E-4	4.29E-2	4.00 (625,9,139,8)
GO:0061448	connective tissue development	2.07E-4	4.47E-2	9.53 (625,8,41,5)
GO:0090092	regulation of transmembrane receptor protein serine/threonine kinase signaling pathway	2.21E-4	4.6E-2	3.26 (625,24,104,13)
GO:0051246	regulation of protein metabolic process	2.22E-4	4.48E-2	1.68 (625,175,100,47)
GO:0048869	cellular developmental process	2.36E-4	4.6E-2	2.23 (625,135,52,25)
GO:0010810	regulation of cell-substrate adhesion	2.36E-4	4.47E-2	2.34 (625,33,162,20)
GO:0051173	positive regulation of nitrogen compound metabolic process	2.41E-4	4.43E-2	1.27 (625,163,334,111)
GO:0051094	positive regulation of developmental process	3.03E-4	5.41E-2	1.94 (625,102,104,33)

Pancreatic Stellate Cells GO analysis of 400 Identified proteins

Cellular Component

GO term	Description	P-value	FDR q-value	Enrichment (N, B, n, b)
GO:0044420	extracellular matrix component	7.43E-11	4.44E-8	20.15 (340,15,9,8)
GO:0005581	collagen trimer	4.97E-7	1.49E-4	10.07 (340,18,15,8)
GO:0005583	fibrillar collagen trimer	6.71E-7	1.34E-4	23.61 (340,8,9,5)
GO:0062023	collagen-containing extracellular matrix	6.83E-7	1.02E-4	2.17 (340,76,70,34)
GO:0031012	extracellular matrix	1.84E-6	2.2E-4	2.10 (340,86,64,34)
GO:0005584	collagen type I trimer	2.08E-4	2.08E-2	85.00 (340,2,4,2)
GO:0044421	extracellular region part	2.39E-4	2.04E-2	1.61 (340,165,46,36)
GO:0005604	basement membrane	2.52E-4	1.89E-2	4.55 (340,21,32,9)
GO:0005576	extracellular region	2.82E-4	1.87E-2	1.39 (340,151,105,65)
GO:0005615	extracellular space	5.57E-4	3.33E-2	1.25 (340,143,181,95)

Molecular Function

GO term	Description	P-value	FDR q-value	Enrichment (N, B, n, b)
GO:0005201	extracellular matrix structural constituent	2.48E-9	1.95E-6	7.35 (340,37,15,12)
GO:0005198	structural molecule activity	1.1E-8	4.31E-6	5.04 (340,59,16,14)
GO:0030020	extracellular matrix structural constituent conferring tensile strength	2.59E-7	6.76E-5	10.67 (340,17,15,8)
GO:0046332	SMAD binding	2.41E-6	4.73E-4	30.91 (340,4,11,4)
GO:0019838	growth factor binding	5.8E-6	9.09E-4	3.67 (340,22,59,14)
GO:0048407	platelet-derived growth factor binding	6.88E-5	8.99E-3	18.89 (340,8,9,4)
GO:0005520	insulin-like growth factor binding	3.35E-4	3.75E-2	4.25 (340,8,70,7)

Biological Processes

GO term	Description	P-value	FDR q-value	Enrichment (N, B, n, b)
GO:0043588	skin development	1.52E-6	7.39E-3	30.22 (340,5,9,4)
GO:0007155	cell adhesion	3.73E-6	9.07E-3	4.74 (340,43,20,12)
GO:0022610	biological adhesion	4.93E-6	8E-3	4.64 (340,44,20,12)
GO:0001501	skeletal system development	7.27E-6	8.85E-3	27.76 (340,7,7,4)
GO:0071230	cellular response to amino acid stimulus	1.05E-5	1.03E-2	27.20 (340,10,5,4)
GO:0071229	cellular response to acid chemical	1.06E-5	8.63E-3	5.75 (340,14,38,9)
GO:0001568	blood vessel development	1.19E-5	8.29E-3	25.19 (340,6,9,4)
GO:0030199	collagen fibril organization	1.45E-5	8.85E-3	15.74 (340,12,9,5)
GO:0043200	response to amino acid	1.7E-5	9.17E-3	24.73 (340,11,5,4)
GO:0001101	response to acid chemical	2.57E-5	1.25E-2	5.37 (340,15,38,9)
GO:0071495	cellular response to endogenous stimulus	3.11E-5	1.38E-2	3.58 (340,38,35,14)
GO:0009653	anatomical structure morphogenesis	7.7E-5	3.12E-2	3.40 (340,65,20,13)
GO:0070887	cellular response to chemical stimulus	7.75E-5	2.9E-2	2.27 (340,86,40,23)
GO:0030198	extracellular matrix organization	2.03E-4	7.04E-2	5.40 (340,36,14,8)
GO:0043589	skin morphogenesis	2.08E-4	6.76E-2	85.00 (340,2,4,2)
GO:1901698	response to nitrogen compound	2.33E-4	7.1E-2	3.53 (340,33,35,12)
GO:0071310	cellular response to organic substance	2.64E-4	7.55E-2	2.36 (340,72,40,20)
GO:1901699	cellular response to nitrogen compound	4.25E-4	1.15E-1	5.95 (340,25,16,7)
GO:1901701	cellular response to oxygen-containing compound	6.06E-4	1.55E-1	2.89 (340,47,35,14)
GO:0043062	extracellular structure organization	7.16E-4	1.74E-1	4.63 (340,42,14,8)
GO:0009719	response to endogenous stimulus	8.17E-4	1.89E-1	2.83 (340,48,35,14)
GO:0050789	regulation of biological process	8.52E-4	1.88E-1	1.18 (340,258,104,93)
GO:0051716	cellular response to stimulus	9.46E-4	2E-1	2.02 (340,106,35,22)
GO:0071417	cellular response to organonitrogen compound	9.68E-4	1.96E-1	11.33 (340,24,5,4)
GO:1901700	response to oxygen-containing compound	9.87E-4	1.92E-1	2.65 (340,55,35,15)

Co Culture condition GO analysis of 540 Identified proteins

Cellular Component

GO term	Description	P-value	FDR q-value	Enrichment (N, B, n, b)
GO:0044421	extracellular region part	1.2E-8	8.55E-6	1.70 (469,213,79,61)
GO:0005576	extracellular region	2.23E-8	7.92E-6	1.52 (469,192,141,88)
GO:0044420	extracellular matrix component	4.83E-8	1.14E-5	17.37 (469,21,9,7)
GO:0031012	extracellular matrix	2.96E-7	5.26E-5	2.28 (469,109,66,35)
GO:0005581	collagen trimer	6.77E-7	9.62E-5	24.68 (469,19,5,5)
GO:0005615	extracellular space	9.52E-7	1.13E-4	1.75 (469,172,81,52)
GO:0005583	fibrillar collagen trimer	1.64E-6	1.66E-4	41.69 (469,9,5,4)
GO:0005584	collagen type I trimer	9.11E-6	8.09E-4	234.50 (469,2,2,2)
GO:0062023	collagen-containing extracellular matrix	2.36E-5	1.86E-3	2.18 (469,98,66,30)
GO:0005604	basement membrane	6.55E-4	4.65E-2	3.01 (469,27,75,13)

Molecular Function

GO term	Description	P-value	FDR q-value	Enrichment (N, B, n, b)
GO:0046332	SMAD binding	3.01E-8	3.05E-5	78.17 (469,4,6,4)
GO:0030020	extracellular matrix structural constituent conferring tensile strength	1.89E-7	9.58E-5	29.31 (469,16,5,5)
GO:0005201	extracellular matrix structural constituent	5.27E-7	1.78E-4	7.99 (469,48,11,9)
GO:0005198	structural molecule activity	1.85E-6	4.69E-4	5.61 (469,76,11,10)
GO:0019838	growth factor binding	3.1E-5	6.28E-3	4.28 (469,28,47,12)
GO:0048407	platelet-derived growth factor binding	2.71E-4	4.57E-2	31.27 (469,9,5,3)
GO:0044389	ubiquitin-like protein ligase binding	2.74E-4	3.96E-2	2.35 (469,18,166,15)
GO:0031625	ubiquitin protein ligase binding	2.74E-4	3.47E-2	2.35 (469,18,166,15)
GO:0050839	cell adhesion molecule binding	4.37E-4	4.92E-2	6.17 (469,38,14,7)
GO:0043168	anion binding	5.55E-4	5.62E-2	2.19 (469,118,40,22)

Biological Processes

GO term	Description	P-value	FDR q-value	Enrichment (N, B, n, b)
GO:0071310	cellular response to organic substance	2E-8	1.16E-4	2.11 (469,87,115,45)
GO:0070887	cellular response to chemical stimulus	1.25E-7	3.62E-4	1.94 (469,103,115,49)
GO:0071229	cellular response to acid chemical	2.48E-7	4.82E-4	6.68 (469,13,54,10)
GO:0071230	cellular response to amino acid stimulus	6.66E-7	9.69E-4	7.72 (469,9,54,8)
GO:0010033	response to organic substance	6.69E-7	7.78E-4	1.97 (469,115,91,44)
GO:0071495	cellular response to endogenous stimulus	8.11E-7	7.86E-4	2.89 (469,42,89,23)
GO:0001101	response to acid chemical	9.03E-7	7.5E-4	6.20 (469,14,54,10)
GO:0009719	response to endogenous stimulus	1.85E-6	1.34E-3	2.31 (469,53,115,30)
GO:0042221	response to chemical	1.86E-6	1.2E-3	1.83 (469,135,91,48)
GO:0043200	response to amino acid	2.98E-6	1.73E-3	37.52 (469,10,5,4)
GO:0009653	anatomical structure morphogenesis	3.75E-6	1.98E-3	3.55 (469,88,24,16)
GO:0051716	cellular response to stimulus	6.81E-6	3.3E-3	1.80 (469,132,93,47)
GO:0043589	skin morphogenesis	9.11E-6	4.08E-3	234.50 (469,2,2,2)
GO:0022610	biological adhesion	2.51E-5	1.04E-2	4.32 (469,62,21,12)
GO:0032502	developmental process	2.63E-5	1.02E-2	1.51 (469,216,86,60)
GO:0030199	collagen fibril organization	2.87E-5	1.04E-2	25.01 (469,15,5,4)
GO:0048856	anatomical structure development	3.4E-5	1.16E-2	1.74 (469,148,82,45)
GO:0043588	skin development	4.1E-5	1.32E-2	46.90 (469,6,5,3)
GO:0034097	response to cytokine	4.34E-5	1.33E-2	2.27 (469,45,115,25)
GO:0001501	skeletal system development	7.46E-5	2.17E-2	20.84 (469,10,9,4)
GO:1901701	cellular response to oxygen-containing compound	8.41E-5	2.33E-2	2.38 (469,52,87,23)
GO:1901699	cellular response to nitrogen compound	8.87E-5	2.35E-2	7.60 (469,27,16,7)
GO:0007155	cell adhesion	1.45E-4	3.67E-2	3.36 (469,61,32,14)
GO:0009887	animal organ morphogenesis	1.47E-4	3.55E-2	5.21 (469,30,27,9)
GO:1901698	response to nitrogen compound	2.58E-4	6.01E-2	3.20 (469,38,54,14)
GO:0009725	response to hormone	2.9E-4	6.48E-2	2.66 (469,23,115,15)
GO:0001568	blood vessel development	3.7E-4	7.96E-2	28.14 (469,10,5,3)
GO:0071417	cellular response to organonitrogen compound	4.12E-4	8.57E-2	3.67 (469,26,54,11)
GO:1901700	response to oxygen-containing compound	4.72E-4	9.46E-2	2.11 (469,64,87,25)
GO:0010243	response to organonitrogen compound	4.82E-4	9.33E-2	3.23 (469,35,54,13)
GO:0040012	regulation of locomotion	5.9E-4	1.11E-1	1.15 (469,89,400,87)
GO:0001957	intramembranous ossification	6.01E-4	1.09E-1	39.08 (469,2,12,2)
GO:0061564	axon development	6.01E-4	1.06E-1	39.08 (469,2,12,2)
GO:0031103	axon regeneration	6.01E-4	1.03E-1	39.08 (469,2,12,2)
GO:0036072	direct ossification	6.01E-4	9.99E-2	39.08 (469,2,12,2)
